# Mobile Health Systems for Community-Based Primary Care: Identifying Controls and Mitigating Privacy Threats

**DOI:** 10.2196/11642

**Published:** 2019-03-20

**Authors:** Leonardo Horn Iwaya, Simone Fischer-Hübner, Rose-Mharie Åhlfeldt, Leonardo A Martucci

**Affiliations:** 1 Privacy and Security (PriSec) Department of Mathematics and Computer Science Karlstad University Karlstad Sweden; 2 School of Informatics University of Skövde Skövde Sweden

**Keywords:** mobile health, mHealth, data security, privacy, data protection, privacy impact assessment, public health

## Abstract

**Background:**

Community-based primary care focuses on health promotion, awareness raising, and illnesses treatment and prevention in individuals, groups, and communities. Community Health Workers (CHWs) are the leading actors in such programs, helping to bridge the gap between the population and the health system. Many mobile health (mHealth) initiatives have been undertaken to empower CHWs and improve the data collection process in the primary care, replacing archaic paper-based approaches. A special category of mHealth apps, known as mHealth Data Collection Systems (MDCSs), is often used for such tasks. These systems process highly sensitive personal health data of entire communities so that a careful consideration about privacy is paramount for any successful deployment. However, the mHealth literature still lacks methodologically rigorous analyses for privacy and data protection.

**Objective:**

In this paper, a Privacy Impact Assessment (PIA) for MDCSs is presented, providing a systematic identification and evaluation of potential privacy risks, particularly emphasizing controls and mitigation strategies to handle negative privacy impacts.

**Methods:**

The privacy analysis follows a systematic methodology for PIAs. As a case study, we adopt the GeoHealth system, a large-scale MDCS used by CHWs in the Family Health Strategy, the Brazilian program for delivering community-based primary care. All the PIA steps were taken on the basis of discussions among the researchers (privacy and security experts). The identification of threats and controls was decided particularly on the basis of literature reviews and working group meetings among the group. Moreover, we also received feedback from specialists in primary care and software developers of other similar MDCSs in Brazil.

**Results:**

The GeoHealth PIA is based on 8 Privacy Principles and 26 Privacy Targets derived from the European General Data Protection Regulation. Associated with that, 22 threat groups with a total of 97 subthreats and 41 recommended controls were identified. Among the main findings, we observed that privacy principles can be enhanced on existing MDCSs with controls for managing consent, transparency, intervenability, and data minimization.

**Conclusions:**

Although there has been significant research that deals with data security issues, attention to privacy in its multiple dimensions is still lacking for MDCSs in general. New systems have the opportunity to incorporate privacy and data protection by design. Existing systems will have to address their privacy issues to comply with new and upcoming data protection regulations. However, further research is still needed to identify feasible and cost-effective solutions.

## Introduction

### Background

Mobile health (mHealth) apps for *health surveys and surveillance* play a crucial role in creating rich data repositories for public health decision-making [1,2]. Apps for health surveys are usually known as mHealth Data Collection Systems (MDCSs), used by Community Health Workers (CHWs), replacing less efficient and less reliable paper-based approaches [3,4]. The CHWs’ main task is to visit families at their homes to provide primary care, but they also carry out surveys, collect the family’s data, and report it to the government. Instead of using paper forms, the CHWs can now use smartphones or tablets for the data collection process.

It is a problem that although mHealth initiatives are developed with a positive and optimistic outlook, there is often little concern for the privacy implications of the app [5]. The existing solutions do not carefully consider privacy and it remains unclear how to deal with the issues inherent to systems of health surveillance. MDCSs are used to collect, process, and share sensitive data (ie, personal health data), making *privacy and security* of paramount importance.

In recent years, much research has focused on the information security aspects of MDCSs [6-9], that is, dealing with the concepts of confidentiality, integrity, and availability, which are commonly addressed by means of security mechanisms for encryption, authentication, secure storage, and access control. Privacy, in turn, stands for the respect of fundamental rights and freedoms of individuals with regard to the processing of personal data. It overlaps with security, especially regarding confidentiality, but many other privacy principles should be addressed (eg, purpose binding, transparency, data minimization, unlikability, intervenability, accountability, and consent)—fundamental differences that are further discussed in this paper. It means that although privacy-preserving systems require strong security, security by itself is not enough.

There are many reasons for enforcing privacy in the primary care context. Privacy is *sine qua non* for achieving high-quality health care [10]. Personal data are collected, processed, and shared in the delivering of health services. Patients (ie, the data subjects) want their information to be used for meaningful purposes, and they want to provide personal data access to health workers so that they can receive proper care. If privacy is not enforced, patients may refrain using the service and/or hold back information, thus preventing health care workers from providing efficient and effective care. The result is inferior quality of health care.

MDCSs are inherently mass surveillance tools. Health care workers may have access to health data of entire communities, so that the privacy impact is amplified. There is a great power imbalance between individuals and the health agencies. Members of underserved communities, typically with less power, face greater risk because of privacy violations [11]. Therefore, it is important to follow *privacy principles* during the design of such systems. Privacy principles have been vastly discussed in the scientific literature and embodied in legal frameworks in various jurisdictions, for example, European General Data Protection Regulation (EU GDPR) [12] and the Brazilian general Bill on the Protection of Personal Data (PLC 53/2018) [13]. Legal frameworks entail compliance, and thus project managers and developers should be prepared to follow such regulations.

Given that, our main research question is the following: *How to design a privacy-aware and secure MDCS?* To answer this question, a Privacy Impact Assessment (PIA) framework is chosen as a strategy for realizing privacy by design. [PIA] *is a systematic process that identifies and evaluates, from the perspectives of all stakeholders, the potential effects on privacy of a project, initiative or proposed system or scheme, and includes a search for ways to avoid or mitigate negative privacy impacts* [14]. PIA comes from the notion of *impact assessment*, defined as *the identification of future consequences of a current or proposed action* [15]. PIAs support a stricter analysis of privacy risks, that is, *effect of uncertainty on privacy* [16]. Each stage of the PIA process builds up on each other, offering not only the risk assessment but also a solid strategy for risk management regarding privacy. In this paper, a PIA is presented using the GeoHealth MDCS [3] as a case study to ground our analysis. As our methodology, the PIA framework proposed by Oetzel et al [17,18] is adopted in this study.

As a result, this paper brings the following contributions: (1) it provides a comprehensive privacy analysis for an MDCS, identifying threats and controls that help project managers and developers solve privacy and data protection issues in their systems, and (2) it shares the experience on how to carry out a PIA for a large-scale mHealth system, as advocated in previous studies [5,19], and it can be seen as an example to other mHealth initiatives. To the best of our knowledge, this is the first thorough privacy analysis for an MDCS. In fact, most mHealth systems neither mention nor appropriately discuss security issues in their systems [20], including privacy.

### Previous Work

This section presents an overview of the previous work in regard to (1) MDCSs, (2) PIA frameworks, and (3) security and privacy of MDCSs. In the sections that follow, various contributions in the area that precedes the current research are described.

### Mobile Health Data Collection Systems Worldwide and in Brazil

Initiatives for replacing paper-based solutions by MDCSs have been increasingly and especially adopted in developing countries [21]. A more recent example is MoTeCH [22,23], employed in Ghana, which empowers nurses and CHWs with a simple mobile app for recording and tracking the care delivered to women and newborns, and it generates management reports mandated by the country’s health authorities. There are also standardized, general purpose tools that help in the task of designing forms and sending them to mobile devices, such as the Magpi framework [24] and the Open Data Kit [25]. Moreover, the World Health Organization together with a group of academic and research institutions and technology partners is developing the Open Smart Register Platform [26], which has been used to empower frontline health workers to electronically register and track the health of their entire client population.

Similarly, many MDCSs have been developed and tested in Brazil. Given the importance of Brazil’s Family Health Strategy (FHS) program for community-based primary care [27], it is natural that various MDCSs focus on the data gathering for the Health Information System for Primary Care (SISAB) database. FHS is one of the most important programs of the Brazilian public health service, *Sistema Único de Saúde*. In the past, the research on MDCSs was mainly developed by research groups inside universities, as it was the case with projects Borboleta [28] and GeoHealth [3].

In this paper, the privacy analysis is particularly grounded on the GeoHealth system. GeoHealth has been targeted in various scientific publications over the years, including work about the design process [8,29], large-scale deployment [3], and CHWs’ experience with the technology [4], which enables us to perform the PIA on the basis of published material, as well as previous first-hand experience with the system.

### Privacy Impact Assessment Frameworks

Many PIA frameworks exist. Some are recommended to a specific jurisdiction and legal framework, whereas others aim for a specific industry sector or for a general methodology. The PIA for Radio Frequency Identification (known as PIA RFID) [18,30] and PIA Smart Grids [31] are examples of sector-specific frameworks. However, the PIA RFID was later generalized in a systematic methodology [17] and it is no longer limited to RFID applications. Other well-known PIA frameworks were proposed by data protection authorities in various countries, such as the British Information Commissioner’s Office (ICO) PIA [32], the Australian Office of the Australian Information Commissioner’s (OAIC) PIA [33], and the French Commission nationale de l'informatique et des libertés’ (CNIL) PIA [34].

More recently, International Organization for Standardization/International Electrotechnical Commission released a standard for PIAs numbered ISO/IEC 29134:2017 [35]. This PIA framework offers as sound methodology with well-defined privacy principles (ISO/IEC 29100), risk identification and evaluation (ISO/IEC 31000 and ISO/IEC 29134), and privacy controls (ISO/IEC 27001 and ISO/IEC 29151). However, it is worth mentioning, that at the ISO/IEC, standards, for example, ISO/IEC 29134 and ISO/IEC 29151, had only been published when this study was already well underway, so they were not chosen as main PIA framework.

In recent years, the systematic PIA methodology [17] also gained more maturity and was endorsed by the Article 29 Data Protection Working Party [36], leading to its adoption for GeoHealth’s PIA. Furthermore, the PIA RFID framework not only provides a robust methodology but it is also accompanied with extensive supplementary material [18,30], openly published and freely accessible since 2011. As far as possible, a parallel among existing PIA frameworks is drawn throughout the paper, given that methods from different PIA frameworks can be combined to better suit the analysis.

### Security and Privacy of Mobile Health Data Collection Systems

Issues regarding *information security* in MDCSs (ie, confidentiality, integrity, and availability) have already been addressed by different authors. For instance, in a study by Cobb et al [9], a range of security threats to MDCSs, that is, Open Data Kit [37], have been identified. In the study [9], the authors detailed a threat modeling exercise on the basis of surveys and interviews with technology experts. Other examples on information security are the works of Gejibo at al [7] and Simplício et al [8] that propose 2 distinct security frameworks for MDCSs. These frameworks are designed to cope with the networking and processing constraints that are inherent to mobile computing. However, both frameworks considerably converge to the same security issues identified in the study by Cobb et al [9].

In addition, regarding mHealth privacy in general, the work of Avancha et al [6] proposes *threat taxonomy* that organizes threats into 3 categories: (1) identity threats, (2) access threats, and (3) disclosure threats. However, privacy is addressed in the study [6] in a rather narrow way. The taxonomy is composed by privacy-related threats, but it essentially overlaps with classical security properties (ie, threats to confidentiality, integrity, and availability). Therefore, if privacy should be considered in a broader dimension, the mHealth threat taxonomy [6] does not contemplate many important Privacy Principles (such as the ones listed in the section “Definition of Privacy Targets”).

Finally, this paper also expands our previous work on GeoHealth’s privacy threat analysis presented in a study by Iwaya et al [38]. On the basis of that, controls are identified and recommended in this paper to mitigate the previously identified threats. In addition, an extensive documentation is provided, enabling research reproducibility of GeoHealth’s PIA and therefore contributing to bridge the knowledge gap between mHealth practitioners and privacy engineers.

## Methods

**Figure 1:**
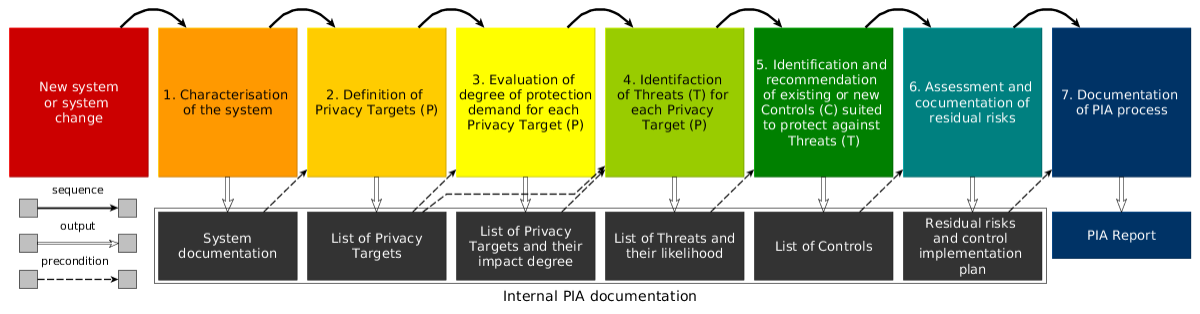
Privacy Impact Assessment (PIA) methodology overview.

This privacy threat analysis follows the PIA framework defined by Oetzel and Spiekermann [17]. In brief, this PIA framework supports project managers and developers to integrate privacy by design in their system development life cycle. The methodology comprises 7 steps, as shown in Figure 1.

Starting with the system characterization in Step 1, the Brazilian GeoHealth MDCS [3,4] is analyzed in the context of previous work on similar solutions [7].

In Step 2, the Privacy Principles and Privacy Targets are defined on the basis of a legal framework. This PIA follows the EU GDPR [12] (enacted in May 2018). This choice is based on 2 reasons: (1) scientifically, the EU GDPR can be considered as state of the art in privacy regulations, and it can be also mapped to the work “A Taxonomy of Privacy [39],” regarded as *“the most complete list of privacy threats* [17].” (2) The current draft of the Brazilian data protection regulation, in a broad way, is akin to the EU GDPR. Even though the health and medical fields often have their own privacy-related regulations, GDPR compliance addresses the privacy problems to a great extent.

In Step 3, the Privacy Targets are evaluated using a degree of protection demand, similar to an impact level (eg, low, medium, and high). During the threat analysis in Step 4, stakeholders identify threats associated to each of the Privacy Targets. All threats are addressed in Step 5 with respective technical and/or nontechnical control measures; residual risk is analyzed, and an implementation plan is specified.

In Step 6, the plan for implementing controls and the remaining residual risk is documented. However, given that GeoHealth has been discontinued and controls cannot be implemented, this step is not performed. For this reason, this PIA can be considered as an *after-the-fact* review, which is still helpful to mHealth practitioners, who might not be particularly keen to publish in-depth public PIA reports about on-going deployments.

As a final outcome of Step 7, this paper can be considered as a “PIA Report” describing the whole analysis, with emphasis on Step 5, “Identification and Recommendation of Controls.” Nonetheless, extensive documentation generated during the PIA process for Steps 1 to 4 is also provided in the form of Appendices.

GeoHealth’s PIA was carried out by our group of researchers with expertise in information security, privacy, and health informatics. Particularly for Steps 3 to 5, the working group meetings were based on evidence from the scientific literature (presented in Section 1). Moreover, 1 of the members participated in the design and development of GeoHealth. Contributions from software developers of other MDCSs as well as specialists in public health and primary care were also received. During the interaction with partners, feedback on our reports and documentation were collected, so that the analysis could be refined.

## Results

This section describes the intermediate results of the PIA process. As explained, Step 5, “Identification and Recommendation of existing or new Controls,” is emphasized in this paper to offer the reader a minimum background. The preceding Steps 1 to 4 are nonetheless summarized, and complete documentation is provided in Multimedia Appendices 1 to 4.

### Characterization of the System

**Figure 2:**
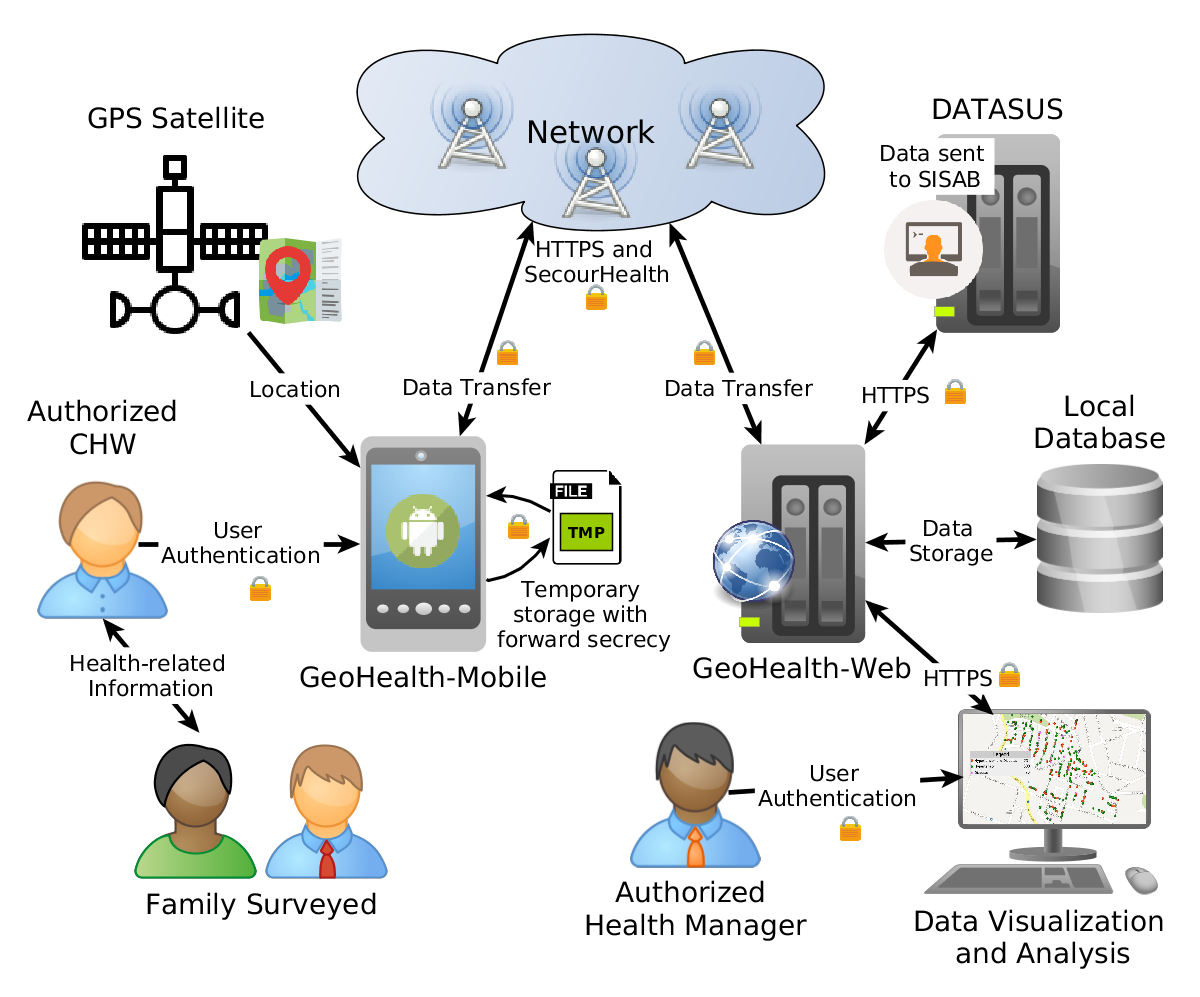
Overview of the GeoHealth actors and their interaction with the system’s components.

GeoHealth is an MDCS tailored for Brazil’s FHS program. It is composed by the GeoHealth-Mobile and the GeoHealth-Web. At the client side, the GeoHealth-Mobile is the Android app that implements all forms used for data collection. At the server side, the GeoHealth-Web implements Web services for receiving and consolidating data as well as for generating reports and exporting data to the national-level system (ie, SISAB/Department of Informatics of the Unified Health System). Figure 2 presents the system architecture, main actors (CHWs, families, physicians, and health managers), and system components.

**Figure 3:**
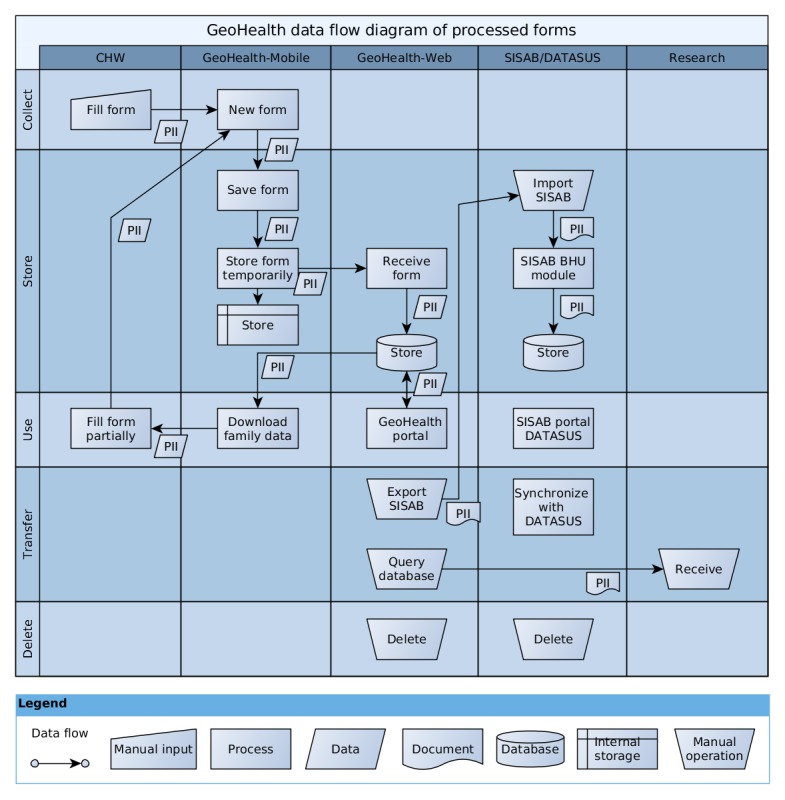
High-level data flow diagram of the GeoHealth environment. Acronyms: Personally Identifiable Information (PII); Basic Health Unit(BHU); Health Information System for Primary Care (SISAB); Department of Informatics of the Unified Health System (DATASUS).

The GeoHealth has been the target of many studies over the last years, so that further information can be found in the original material [3,4,8,29], as well as in a comprehensive description in Multimedia Appendix 1 [40,41]. For the readers’ convenience, the data flow diagram presented in Figure 3 shows how personal information is handled by the different subprocesses.

### Definition of Privacy Targets

After the system characterization, the next step is to determine the privacy principles that will be the basis of the design of our system. In the study by Oetzel and Spiekermann [17], the authors distinguish between *privacy principles* and *privacy targets*. Both terms were not explicitly defined, but privacy principles can be considered as a fundamental, primary, or general rule derived from the existing legal frameworks [12,42]. However, as explained by the study [17], these legal privacy principles must be translated into concrete, auditable, and functionally enforceable Privacy Targets and subsequent system functions. Furthermore, Privacy Targets should be formulated as action items, just like in widely accepted modeling techniques such as Unified Modeling Language and Architecture of Integrated Information Systems.

Textbox 1 presents a list of privacy principles and respective Privacy Targets derived from the European General Data Protection Regulation and originally conceived by Oetzel and Spiekermann [17,38]. Although this list was used as a baseline for this PIA, all Privacy Targets were reviewed in terms of applicability, meaning, and exhaustiveness in the context of GeoHealth. As a result of this revision, the principle *P5-Intervenability* was added and the targets that were previously listed under *P4 - Access Right of Data Subject* were moved to this new category (ie, P5.1, P5.2, and P5.3). Thus, now there is a clear distinction between data subject access (transparency) and intervenability. Furthermore, new Privacy Targets P4.2 and P5.4 were proposed and added to the list.

**List of Privacy Principles and Privacy Targets.**

P1-Quality of data processing

- P1.1 - Ensuring processing in a lawful, fair, and transparent manner
- P1.2 - Ensuring processing only for legitimate purposes
- P1.3 - Providing purpose specification
- P1.4 - Ensuring limited processing for specified purpose
- P1.5 - Ensuring data avoidance
- P1.6 - Ensuring data minimization
- P1.7 - Ensuring data quality, accuracy, and integrity
- P1.8 - Ensuring limited storage

P2-Processing lawfulness (and informed consent)

- P2.1 - Ensuring legitimacy of personal data processing
- P2.2 - Ensuring legitimacy of sensitive personal data processing

P3-Information right of data subject (ex ante transparency)

- P3.1 - Providing adequate information in cases of direct collection of data from the data subject
- P3.2 - Providing adequate information where data has not been obtained directly from the data subject (eg, from third parties)

P4-Access right of data subject (ex post transparency)

- P4.1 - Facilitating the provision of information about processed data and purpose
- P4.2 - Facilitating the provision of an (electronic) copy of data

P5-Intervenability

- P5.1 - Facilitating the rectification, erasure, or blocking of data
- P5.2 - Facilitating the portability of data
- P5.3 - Facilitating the notification to third parties about rectification, erasure, and blocking of data
- P5.4 - Providing the ability to withdraw consent

P6-Data subject’s right to object

- P6.1 - Facilitating the objection to the processing of personal data
- P6.2 - Facilitating the objection to direct marketing activities
- P6.3 - Facilitating the objection to disclosure of data to third parties
- P6.4 - Facilitating the objection to decisions that are solely based on automated processing of data
- P6.5 - Facilitating the data subject’s right to dispute the correctness of machine conclusions

P7-Security of processing

- P7.1 - Ensuring the confidentiality, integrity, and availability of personal data storage, processing, and transmission
- P7.2 - Ensuring the detection of personal data breaches and their communication to data subjects

P8-Accountability

- P8.1 - Ensuring the accountability of personal data storage, processing, and transmission

### Evaluation of the Degree of Protection Demand for Each Privacy Target

Each of the listed Privacy Targets was put in context and further evaluated. In this step of the PIA, Privacy Targets were ranked and priorities for the GeoHealth’s privacy architecture were identified. To determine the right level of protection that each Privacy Target demands, a potential damage scenario had to be considered, that is, using the *“feared events”* technique by asking, *“What would happen if...?”* Every Privacy Target was challenged by its potential damage in case of noncompliance. Furthermore, the damage had to be considered from 2 perspectives: the system operator (eg, loss of reputation and financial penalties) and its customer (eg, social embarrassment, financial losses, and jeopardize personal freedom).

A qualitative approach is used because privacy breaches are often “softer” or intangible (eg, hurt feelings, discredit, blackmail, and even death) rather than something with a specific monetary value (eg, a computer system or asset). Being qualitative is a major difference of the PIA methodology when compared with more quantitative asset-driven evaluations for security assessments. That is, assets such as data, software, and hardware are easier to quantify, such as loss and cost, whereas reputation, embarrassment, and harm to people’s rights and freedoms are not. This part of the PIA process is detailed in Multimedia Appendix 2.

### Identification of Threats for Each Privacy Target

For each Privacy Target, the threats that could impede us from achieving each target are systematically identified. A threat is essentially a noncompliance with the relevant privacy laws and standards that emerge from multiple sources, such as the lack of training and privacy awareness, inappropriate use, privacy-preserving technologies, or absence of privacy management and governance practices [17].

The identification of privacy threats for GeoHealth was presented as part of our previous work [38]. Further details can be also found in Multimedia Appendix 3. In summary, this threat analysis was built upon existing threats analyses for mHealth in general [6] or specifically for MDCSs [7,9], as well as privacy threats (for RFID) found in the study by Oetzel et al [18]. Thus, this threat analysis is not only based on the assessment of privacy experts but also on existing scientific literature, from which threats were reviewed and compiled.

As a result, 22 groups of threats and a total of 97 subthreats are identified. Threats can range greatly, jeopardizing principles such as data quality, processing legitimacy, informed consent, right to information, right to access, right to object, data security, and accountability. The threats were also classified as “likely” (n=86) or “unlikely” (n=11) to happen, enabling us to assertively assign controls.

### Identification and Recommendation of Controls to Protect Against Threats

As a point of departure, a list of possible controls presented in a study by Oetzel et al [30] is used, combined with the security controls proposed in previous studies [7-9,43]. The final list is composed by 41 recommended controls (Table 1, further details in Multimedia Appendix 4) to cope with the identified privacy threats. According to the methodology, each control has up to 3 levels of rigor: (1) satisfactory, (2) strong, and (3) very strong. During the process of assigning controls for each threat, a level of rigor is also chosen, defining how extensive the control should be, which is likely costlier and more difficult. The level of rigor should match the previously defined level of protection demand determined in the section “Evaluation of Degree of Protection Demand for each Privacy Target.” However, for the GeoHealth case study, all the threats are linked to at least one or more Privacy Targets with a “high” level of protection demand. Therefore, all controls in the consolidate list only need to be described for a “very strong” level of rigor (see Multimedia Appendix 4). Table 2 shows the association of controls to the identified threats.

**Table 1:** Consolidated list of controls. The detailed description of all controls can be found in Multimedia Appendix 4.

Control codes and short descriptions	Done?
C1.1 Service description	—^a^
C1.2 Information accessibility	—
C1.3 Language/semantics of information	—
C1.4 Information timeliness	—
C1.5 Privacy statement	—
C1.7 Purpose specification	—
C1.8 Ensuring limited data processing	—
C1.9 Ensuring purpose related processing	—
C1.10 Ensuring data minimization	Partly
C1.12 Ensuring personal data quality	Yes
C1.14 Ensuring data accuracy	Yes
C1.15 Enabling data deletion	—
C3.1 Obtaining data subjects’ explicit consent	Partly
C4.1 Providing data processing information	Partly
C4.2 Providing information on third party information processing	—
C5.1 Informing data subjects about data processing	—
C5.3 Handling data subjects change requests	—
C5.4 Providing data export functionality	—
C5.5 Handling exemptions and derogations	—
C6.1 Notifying data subjects about sharing practices	—
C6.2 Handling objections (to automated decisions)	—
C7.1 Ensuring data subject authentication	—
C7.2 Ensuring staff authentication	Yes
C7.3 Ensuring device authentication	Partly
C7.4 Providing usable authentication	Partly
C7.5 Logging access to personal data	Yes
C7.6 Performing regular privacy audits	—
C7.7 Ensuring data anonymization	Partly
C7.8 Providing confidential communication	Yes
C7.9 Providing usable access control	—
C7.10 Ensuring secure storage	Yes
C7.11 Ensuring physical security of infrastructure	—
C7.12 Providing locked down devices	Yes
C7.13 Providing memory wipe	—
C7.14 Enabling offline authentication	Yes
C7.15 Network monitoring	—
C7.16 Preventing denial-of-service attacks	—
C7.17 Handling security incidents	—
C8.1 Demonstrate data privacy accountability	—
C8.2 Notification of authority	—
C8.3 Notification of data subjects	—

^a^The control was not implemented.

**Table 2:** Threat groups and associated controls. The detailed description of all subthreats can be found in Multimedia Appendix 3.

Threat Group	Description	Controls
T1.1-T1.5	Lack of transparency, missing or insufficient service information	C1.1, C1.2, C1.3, C1.4, and C6.2
T1.6-T1.10	Lack of transparency, missing or insufficient privacy statement	C1.5
T1.11-T1.18	Unspecified and unlimited purpose	C1.7, C1.8, C1.9, and C1.10
T1.19-T1.24	Collection and/or combination of data exceeding purpose	C1.8, C1.9, and C1.10
T1.25-T1.30	Missing quality assurance of data	C1.12, C1.14, and C7.1
T1.31-T1.34	Unlimited data storage	C1.15 and C1.10
T2.1-T2.8	Invalidation or nonexistence of consent	C3.1 and C5.5
T3.1-T3.5	No or insufficient information concerning collection of data from the data subject	C4.1, C4.2, and C5.1
T4.1-T4.4	Inability to provide individualized information about processed data and purpose	C5.1, C7.1, and C7.5
T5.1-T5.6	Inability to rectify, erase, or block individual data	C1.15, C5.3, C7.1, C7.5, and
T5.7	Inability to notify third parties about rectification, erasure and blocking of individual data	C5.3
T5.8-T5.10	Inability to support data portability for individual data	C5.4
T6.1	Inability to allow objection to the processing of personal data	C6.1 and C6.2
T6.2-T6.5	Inability to allow objection to the disclosure of data to third parties	C4.2, C6.1, and C6.2
T6.6	Inability to allow objection to being subject to decisions that are solely based on automated processing of data	C6.2
T7.1-T7.3	Identity threats, misuse and leakage of data subject identities [21]	C7.1, C7.5, C7.6, C7.7, and C7.8
T7.4-T7.11	Access threats, unauthorized access and modification of PHI^a^ or PHR^b^ [21]	C5.5, C7.2, C7.5, C7.6, C7.9, C7.10, and C7.11
T7.12-T7.19	Disclosure threats, unauthorised disclosure and data leaks of PII^c^ and PHI [21]	C7.2, C7.3, C7.4, C7.5, C7.6, C7.8, C7.10, C7.12, and C7.13
T7.20-T7.21	Denial-of-service threats [22,24]	C7.3, C7.10, C7.14, C7.15, and C7.16
T7.22-T7.24	Inability to detect personal data breaches and communicate them to data subjects	C7.5, C7.6, C7.17, C8.2, and C8.3
T8.1-T8.2	Lack of accountability of personal data storage, processing, and transmission	C7.6, C8.1, and C8.4
T8.3-T8.6	Noncompliance with notification requirements	C8.2 and C8.4

^a^PHI: protected health information.

^b^PHR: personal health record.

^c^PII: personally identifiable information.

^c^Note that each group of threats has a number of more specific subthreats (eg, T1.1, T1.2, and T1.3). The technical or organizational controls (listed in Table 1) can then be associated to 1 or more subthreats.

## Discussion

### Principal Findings

In summary, GeoHealth’s PIA is based on 8 Privacy Principles and 26 Privacy Targets derived from the EU GDPR. Associated to that, 22 threat groups with a total of 97 subthreats and 41 recommended controls are identified. Thus, offering a sound privacy analysis for a large-scale MDCS.

This research shows that the literature mostly focuses on the information security issues, solving only a fraction of the problem, that is, (P6) Security of Data. Currently, there is a lack of contributions on how to engineer privacy not only in MDCSs but also for the area of mHealth in general [5,19]. Our PIA helps to bridge this gap by exposing the problems and providing controls (see Multimedia Appendix 4). On the basis of this PIA, engineers have a clearer path toward solving the privacy issues and ideally being able to address them at the very early stages of the design process, when changes are often simpler and less costly.

In addition, the consolidated list of controls, in Table 1, also makes it clear that privacy cannot be dealt only with technical measures. In fact, most controls required a mixed approach of technical and organizational procedures that should be put in place to achieve privacy and data protection. One way of doing this is to integrate the organizational procedures related to privacy in an information security management system to facilitate for organizations and make the processes for both information security and privacy more efficient. This could be a task for further research.

Another important finding from the PIA is that some privacy issues are more challenging, requiring major changes on the existing MDCSs. However, it is not within the scope of a PIA to provide complete solutions to solve such challenges but rather to make them explicit. The main privacy challenges for MDCSs include the following: (1) individualized access to personal data to provide transparency and intervenability, (2) obtaining and handling explicit informed consent from data subjects and allowing consent withdrawal, (3) defining measures to object processing and allow data blocking or deletion, (4) employ security mechanisms, and (5) utilize appropriate anonymization techniques for data sharing. In the sections that follow, the discussion on each of these privacy challenges is expanded.

### Transparency and Intervenability

Among the main findings, it is noticeable that existing MDCSs particularly fall short with respect to GDPR principles of *transparency and intervenability*, that is, (P1) Quality of Data Processing, (P4) Access Right of Data Subject, and (P5) Intervenability. In brief, MDCSs do not consider the data subjects’ personalized access to their data in electronic form, and in fairness, they were designed to be accessed only by CHWs and medical staff. However, it is worth mentioning that to achieve GDPR compliance, nonelectronic access is sufficient. Nonetheless, as a matter of enhanced privacy by design (and not purely compliance), major redesign is required to add data subjects as system users and to support interaction with a personalized interface (eg, a privacy dashboard), somewhat similar to existing online medical records [44]. In this line, MDCSs would benefit from emerging Transparency-Enhancing Tools [45] that help to raise privacy awareness among data subjects by allowing them to know about the data that are collected and processed about them and the potential privacy risks (eg, discriminatory profiling, data breaches, and leaks). However, such changes greatly expand the system’s attack surface (ie, a new category of users with access rights) and increase the costs of software development and underlying infrastructure. Therefore, the redesign of MDCSs requires further feasibility studies, especially for projects running in low- and middle-income countries.

### Informed Consent

*Explicit informed consent* (ie, a signed written statement) [46] also has some particularities. Consent is a well-known requisite for providing medical treatment. In MDCSs, the consent is given for the processing of personal data. It refers to the data collection, processing, and access rights to the data and for the purpose stated, that is, it is about technologies and systems. Just as importantly, consent revocation needs to be as easily made as giving consent. As CHWs use smartphones for data collection, it is difficult for data subjects to withdraw their consent later, as they do not have direct computer access. Asking to revoke consent via telephone is not an easy solution either, as the data subjects must be properly identified first. There should also be routines for allowing to revoke the consent only for selected purposes (eg, a partial agreement, as there should be opt-in options for each purpose). Existing literature on MDCSs does not discuss opt-ins, but there are guidelines to help project managers [46].

On the other hand, consent is not the only lawful basis for personal data processing. Public health and social care can also rely on legitimate interests and the performance of a public task as justifications for the processing of personal data. However, some MDCSs can also be used for secondary purposes, which should be made optional to data subjects. For instance, linking the data subjects’ personal data to other electronic health records or disclosing it for research and statistics outside the public health sphere. However, there is an immense power asymmetry between the public health system and the individuals. When the majority of the population relies uniquely on the public system, there is never really a free choice. That is, if data subjects are coerced or if there is a threat of disadvantage (eg, no health care) the consent can be rendered invalid.

### Data Objection and Deletion

Features for *automated data deletion* are also missing in the existing MDCSs. That may be seen as a technicality that is just not explored in the MDCS literature, but it is associated with the well-known right to be forgotten and data minimization principle. For MDCSs, families may also change their address or move to other communities, which would require formal procedures for automated deletion, as well as *data portability* (ie, to send the family’s data to another health unit). Data subjects may also require deletion or blocking of sensitive data that can impact their privacy. More importantly, medical conditions with strong genetic components can disclose information about the patient’s relatives, that is, impacting other people’s privacy. Individual privacy preferences pose challenges for executing data subject rights, as the data may refer to multiple data subjects, who all may have rights by different interest (eg, one may want the data to be deleted whereas the other would like data to be preserved). Routines are needed to handle such disputes and situations. In some cases, it may be possible to pseudonymize the identity of the person that wants his or her data to be deleted (eg, in case of infections), whereas in case of genetic relations, it may not currently be possible.

However, it is essential to know that medical information related to medical conditions and procedures cannot be deleted even if the data subject requests, that is, with respect to legal aspects of medical records alterations. Instead, because this is sensitive information, the protection mechanisms are even more important.

### Security Mechanisms

Security frameworks specifically designed for MDCSs have already been proposed [7,8]. In brief, MDCSs need a Key Management Mechanism to provide Authentication and Key Exchange among parties (user’s mobile and app server). Authentication protocols and key derivation schemes for MDCSs usually rely on symmetric cryptography, using password authentication. These protocols should also give support for online and offline user authentication so that users are not limited because of the lack of network connectivity or coverage. Other mechanisms should cope with the confidentiality of stored and in-transit data by means of encryption schemes for secure storage and transmission.

### Anonymization

MDCSs also support the creation of rich repositories of health-related data needed for the planning, implementation, and evaluation of public health practice. These datasets are often used for secondary purposes by government agencies, researchers, and academics. In such cases, the data should be anonymized, that is, to protect privacy by making a number of data transformations so that individuals whom the data describe remain anonymous. The anonymization process can have variable degrees of robustness [47], depending on how likely is to (1) single out an individual in the dataset, (2) link records concerning the same individual, or (3) infer the value of 1 attribute on the basis of other values. In essence, all these circumstances should be avoided, resulting in an anonymized dataset. Anonymized data are not considered personal data; therefore, data privacy laws no longer apply. Although the literature on data anonymization is vast, fully anonymized datasets are difficult or even impossible to achieve. The Working Party 29 has already expressed an opinion on this matter [47].

### Limitations

Although this PIA had been carefully designed and conducted, limitations of the research must be acknowledged. First, regarding methodological aspects, a parallel with other approaches for risk assessment can be drawn. That is, PIAs, as any risk assessment methodology, have inherent limitations [48]: (1) the estimation of risk is never complete in the mathematical sense, (2) a complete set of undesired events (threats) is never known, (3) no way is provided to deal with unknown vulnerabilities and attacks, and (4) continuous revision is always required. PIAs are not different. PIAs should be periodically reviewed, whenever assumptions change or when new threats are unveiled. Nonetheless, by performing a PIA and implementing controls, organizations demonstrate that they are tackling privacy and data protection issues due diligence.

Second, although the PIA RFID framework [17] offers a sound methodology, there are other PIA frameworks that are already published (eg, OAIC’s PIA, British ICO’s PIA Handbook, CNIL’s PIA manual, and ISO/IEC 29134). Some approaches are more streamlined (eg, OAIC’s PIA and British ICO’s PIA Handbook) and consequently not so grounded on technical standards (eg, PIA RFID framework and ISO/IEC 29134). Moreover, as mentioned before, the chosen PIA framework also utilizes a qualitative approach for risk assessment, which differs from quantitative and asset-driven approaches that are more common for security risk analysis. A comparison study of PIA frameworks is outside the scope of this paper, but it may be beneficial to the community.

Third, a few remarks can be also made about the way in which the PIA was conducted. Ideally, the PIA should be carried out in consultation with all relevant stakeholders (eg, developers, health care workers, data subjects and/or representatives, and policy-makers). The PIA was conducted by the authors who come from multiple disciplines (information security, medical informatics, and law) and have first-hand experiences with MDCSs. Besides, input and feedback were provided by software engineers from 2 industry partners with experience in developing MDCS. In conducting this PIA, the authors adopted the role of the data subjects to articulate their perspectives and advocate for their privacy. Two of the authors are members of privacy interest organizations and/or former members of the advisory board of the Swedish Data Protection Commissioner. The authors are therefore used to taking the perspective of data subjects and are more experienced in analyzing privacy issues on behalf of the data subjects than most laypersons. Nonetheless, especially after the MDCS is rolled out, it is recommended to consult the families enrolled in the primary care programs directly and gather their perspectives and concerns regarding privacy on the basis of their personal experiences for conducting another iteration of the PIA.

### Conclusions

CHWs are crucial in the Brazilian health care scenario, and empowering them with relevant tools can revolutionize the delivery of community-based primary care. MDCSs are proven effective tools to support the activities of CHWs in Brazil [3] and around the world [1]. However, solving privacy and data protection issues is imperative for the successful deployment of such systems. In fact, as advocated in previous studies [5,19], a careful look into privacy is still notably lacking in many mHealth projects and initiatives. This paper offers a full PIA for the GeoHealth MDCS aiming to unveil the privacy pitfalls that large-scale mHealth systems may have. Our results show that important privacy principles could be further enhanced, such as data minimization, obtaining consent, enabling data processing transparency, and intervenability. In fairness, existing research may not primarily account for privacy, as privacy-preserving features are considered as nonfunctional requirements or even because such considerations are beyond the scope of many papers. Nonetheless, systems that are already deployed, especially in health care, should be compliant with the principles of privacy by design.

Besides, as discussed, the literature on Privacy-Enhancing Technologies (PETs) already has a range of mechanisms for consent management, transparency, and intervenability. Therefore, the future work in MDCSs involves the evaluation of suitable PETs mainly accounting for the implementation of technical controls as well as to migrate organizational controls with information security management processes.

## Appendix

Multimedia Appendix 1 Characterization of the System.

Multimedia Appendix 2 Evaluation of degree of protection demand.

Multimedia Appendix 3 Identification of privacy threats.

Multimedia Appendix 4 Technical and non-technical controls.

## Abbreviations

CHWs: Community Health Workers
CNIL: Commission nationale de l'informatique et des libertés
EU GDPR: European General Data Protection Regulation
FHS: Family Health Strategy
ICO: Information Commissioner’s Office
IEC: International Electrotechnical Commission
ISO: International Organization for Standardization
MDCS: Mobile Health Data Collection System
mHealth: mobile health
OAIC: Office of the Australian Information Commissioner
ODK: Open Data Kit
PETs: Privacy-Enhancing Technologies
PIA: Privacy Impact Assessment
RFID: Radio Frequency Identification
SISAB: Health Information System for Primary Care (Sistema de Informação em Saúde para a Atenção Básica)

## References

[1] World Health Organization (2011). Global Observatory for eHealth.

[2] Pencarrick HC, Meagher N, McGrail KM (2013). Privacy by design at population data BC: a case study describing the technical, administrative, and physical controls for privacy-sensitive secondary use of personal information for research in the public interest. J Am Med Inform Assoc.

[3] Sá JH, Rebelo MS, Brentani A, Grisi SJ, Iwaya LH, Simplício MA, Carvalho TC, Gutierrez MA (2016). Georeferenced and secure mobile health system for large scale data collection in primary care. Int J Med Inform.

[4] Schoen J, Mallett JW, Grossman-Kahn R, Brentani A, Kaselitz E, Heisler M (2017). Perspectives and experiences of community health workers in Brazilian primary care centers using m-health tools in home visits with community members. Hum Resour Health.

[5] Crowe A (2014). Taking Privacy and Data Protection Seriously in M4D Initiatives. Proceedings of the 4th International Conference on M4D Mobile Communication for Development: M4D 2014, General Tracks.

[6] Avancha S, Baxi A, Kotz D (2012). Privacy in mobile technology for personal healthcare. ACM Comput Surv.

[7] Gejibo S, Mancini F, Mughal K, Sasan A (2015). Mobile data collection: a security perspective. Mobile Health: A Technology Road Map.

[8] Simplício MA, Iwaya LH, Barros BM, Carvalho TC, Näslund M (2015). SecourHealth: a delay-tolerant security framework for mobile health data collection. IEEE Biomed Health Inform.

[9] Cobb C, Sudar S, Reiter N, Anderson R, Roesner F, Kohno T (2018). Computer security for data collection technologies. Dev Eng.

[10] Cooper T (2007). Healthcare Information and Management Systems Society (HIMSS).

[11] Gangadharan SP (2015). The downside of digital inclusion: expectations and experiences of privacy and surveillance among marginal Internet users. New Media Soc.

[12] European Commission (2016). EUR-Lex.

[13] (2018). Senado Federal.

[14] Clarke R (2011). An evaluation of privacy impact assessment guidance documents. International Data Privacy Law.

[15] Clarke R (2009). Privacy impact assessment: its origins and development. Comput Law Secur Rev.

[16] ISO (2011). International Organization for Standardization.

[17] Oetzel MC, Spiekermann S (2013). A systematic methodology for privacy impact assessments: a design science approach. Eur J Inf Syst.

[18] Oetzel MC, Spiekermann S, Grüning I, Kelter H, Mull S (2011). [Federal Office for Information Security (BSI)].

[19] TrustLaw (2013). TrustLaw Connect.

[20] Iwaya LH, Gomes MA, Simplício MA, Carvalho TC, Dominicini CK, Sakuragui RR, Rebelo MS, Gutierrez MA, Näslund M, Håkansson P (2013). Mobile health in emerging countries: a survey of research initiatives in Brazil. Int J Med Inform.

[21] Shao D (2018). Malmö University.

[22] Grameem Foundation (2012). Grameem Foundation.

[23] LeFevre AE, Mohan D, Hutchful D, Jennings L, Mehl G, Labrique A, Romano K, Moorthy A (2017). Mobile Technology for Community Health in Ghana: what happens when technical functionality threatens the effectiveness of digital health programs?. BMC Med Inform Decis Mak.

[24] Magpi Magpi.

[25] Anokwa Y, Hartung C, Brunette W, Borriello G, Lerer A (2009). Open source data collection in the developing world. Computer.

[26] OpenSRP Open Smart Register Platform.

[27] Macinko J, Harris MJ (2015). Brazil's family health strategy--delivering community-based primary care in a universal health system. N Engl J Med.

[28] Correia R, Kon F, Kon R (2008). Borboleta: A mobile telehealth system for primary homecare.

[29] Sá JH, Rebelo M, Brentani A, Grisi S, Gutierrez MA (2012). GeoHealth: a georeferenced system for health data analysis in primary care. IEEE Latin Am Trans.

[30] Oetzel M, Spiekermann S, Grüning I, Kelter H, Mull S (2011). Bundesamt für Sicherheit in der Informationstechnik (BSI).

[31] EU Commission (2014). European Commission.

[32] Information Commissioner's Office (2014). Information Commissioner's Office.

[33] Office of the Australian Information Commissioner (2014). Office of the Australian Information Commissioner.

[34] CNIL (2018). [National Commission for Informatics and Liberties].

[35] ISO (2017). International Organization for Standardization.

[36] Wright D (2012). The state of the art in privacy impact assessment. Comput Law Secur Rev.

[37] (2018). Open Data Kit.

[38] Iwaya LH, Fischer-Hübner S, Åhlfeldt RM, Martucci L (2018). mHealth: a Privacy Threat Analysis for Public Health Surveillance Systems. IEEE 31st International Symposium on Computer-Based Medical Systems (CBMS).

[39] Solove DJ (2006). A taxonomy of privacy. Univ PA Law Rev.

[40] SISAB (2018). [Department of Primary Care].

[41] DATASUS (2018). [IT Department of SUS].

[42] EU Commission (1995). EUR-Lex.

[43] Kotz D (2011). A threat taxonomy for mHealth privacy.

[44] Rexhepi H, Åhlfeldt RM, Cajander Å, Huvila I (2016). Cancer patients' attitudes and experiences of online access to their electronic medical records: A qualitative study. Health Informatics J.

[45] Murmann P, Fischer-Hübner S (2017). Tools for achieving usable ex post transparency: a survey. IEEE Access.

[46] EU Commission (2017). European Commission.

[47] WP29 (2014). European Commission.

[48] Cherdantseva Y, Burnap P, Blyth A, Eden P, Jones K, Soulsby H, Stoddart K (2016). A review of cyber security risk assessment methods for SCADA systems. Comput Secur.

