# Supplementary material for: Mobile Health Systems for Community-Based Primary Care: Identifying Controls and Mitigating Privacy Threats
Source: JMIR Mhealth Uhealth. 2019 Mar 20;7(3):e11642. doi: 10.2196/11642 (PMC6446152; doi:10.2196/11642)
Supplement: Multimedia Appendix 1 [file mhealth_v7i3e11642_app1.pdf]

Multimedia Appendix

This is a Multimedia Appendix to a full manuscript published in the JMIR mHealth and uHealth.

Appendix 1

Characterisation of the System

The system has to be described in a comprehensive and detailed way so that potential privacy problems can be detected. To do so, we adopt the four views strategy, as recommended in the methodology:

1) *System view*: application and system components, hardware, software, internal and external interfaces, network topology;
2) *Functional view*: generic business processes, detailed use cases, roles and users, technical controls;
3) *Data view*: categories of processed data, data flow diagrams of internal and external flows, including actors and data types;
4) *Physical environment view*: physical security and operational controls such as backup and contingency.

The main reference about GeoHealth [3] is not sufficiently detailed nor presents the system through multiple views. Thus, in this section we further detail the GeoHealth's documentation in order to perform a reliable PIA.

System View

GeoHealth follows a client-server model composed by the GeoHealth-Mobile (client) and the GeoHealth-Web (server). Figure 2 gives an overview of the interaction between GeoHealth actors (CHAs, physicians and health managers) and system components.

GeoHealth-Mobile

GeoHealth-Mobile is an application for Android smartphones. The application is used by Community Health Agents (CHAs) that are collecting data. The main smartphone features used by the GeoHealth-Mobile are the IO interface to fill-up the forms, networking (3G and 4G) for data communication, camera for tracking community evolution, and GPS for georeferencing data.

GeoHealth-Web

GeoHealth-Web is a system (Apache Tomcat) that runs in the web server and has a central database (MySQL) storing all the information collected by CHAs using the GeoHealth-Mobile. Authorised access to the information is granted to CHAs and medical staff according to the region that they were designated and families that are under their supervision. This information should be however exported to the SISAB [47], formerly known as SIAB (Health Information System for Primary Care) that aggregates information at national level (used mainly for health managers and researchers for statistical analysis). Data communication between GeoHealth-Mobile and GeoHealth-Web passes through the Mobile Network Operator (MNO).

GeoHealth-Web exports data to the SIAB module installed in a local server, so that the SIAB application can communicate through the Internet with the DATASUS (Department of Informatics of the Brazilian's Unified Health System [48]).

Functional View

Overall Goals and Business Process

The Unified Health System (SUS) is Brazil's publicly funded health care system. Family Health Strategy (FHS) is one of the national public health programs in Brazil, which implements a national policy for primary care settings with the aim of substituting part of the traditional model of primary care based on medical specialists. Its main focus is on families instead of individuals, and it is organised around multidisciplinary Family Health Teams (FHTs), formed by a core of professionals such as physicians, nurses, dentists, psychologists and social workers, as well as Community Health Agents (CHAs). CHA is the title of a specific lay health care worker developed in Brazil by way of PACS (Program of Community Health Workers) in 1991 as part of the construction of the Brazilian UHS established by Constitutional rule in 1988. The agents' primary task is gathering information on the health status of a small community by means of a close relationship with it. In its design, the agent should be a neighbourhood resident to be selected on the basis of a good relationship with his neighbours for a fulltime employment. Every group of agents is supervised by a physician or nurse of the health clinic, and home visits are conducted in the coverage area of a Basic Health Unit (BHU), thus producing information that can assess the main health problems of his community.

As part of their work, CHAs have to periodically fill forms for every family that is under their coverage area. GeoHealth was designed to replace the paper-based data collection process by an electronic one. Thus, reducing the amount of paper work and making the whole process more efficient and reliable.

GeoHealth's Detailed Use Case

GeoHealth's main users are the CHAs and medical staff working at the basic health units distributed across the country. Customarily, the GeoHealth-Mobile is used by CHAs while the GeoHealth-Web is used by the staff for data analysis and health promotion in their respective coverage areas. At the same time the GeoHealth's database serves as a rich repository for public health surveillance and health-related research. In this way, we can decompose the system in four different sub-processes: (a) Data Collection & Family Care; (b) Data Analysis & Health Promotion; (c) Public Health Surveillance; (d) Health-related Research.

Here, we are mainly focused on detailing the sub-processes (a) and (b), basically because we have full control over the design of these sub-processes and they are the most critical for the FHS. Besides, we present in this section a list of the most representative stakeholders in the GeoHealth environment.

Data Collection & Family Care – Every month the CHAs visit families at their homes in order to provide primary care and collect a wide range of health-related information. This is the most important activity to operationalize the FHS. During the visit, the CHAs talk to the family members and use a set of forms to survey their

health and living conditions. Table 4 presents a summary about the main forms that feed the SIAB. All the information is securely stored in the mobile phone, and then, sent to the server as soon as network connectivity is available. All the information is sent encrypted from the mobile device and decrypted only when it reaches the server. So, the server decrypts all the data and consolidates it in a central database. CHAs and medical staff have access to this database. They can upload forms and download information about the families that they work with using the GeoHealth-Mobile or the GeoHealth-Web (via browser). All the forms about the families are monthly synchronized with the SIAB database. Forms can be edited while they are being filled by the CHAs, but as soon as they are complete and sent, neither CHAs or medical staff can edited the information (only the database administrator can do that). CHAs can also download basic information about families to avoid repetitive filling of data (i.e. to start with a partially filled forms).

Table 4. Examples of standardized forms* of the Family Health Strategy (FHS).

Form	Description
A	Family registration form. Contains information about household characteristics (e.g. address, sanitation and dwelling conditions, number of residents, self-reported chronic diseases or pregnancy, and immunization status), and about family members (e.g. age, sex, education level, and occupation).
B	A set of forms for monitoring the FHS priority health conditions, namely pregnancy, hypertension, diabetes, tuberculosis and leprosy.
C	Open form used specially for monitoring children from 0 to 60 months, including information such as age, weight, breast-feeding status, hospitalizations, etc.
D	Daily report of FHTs activities and procedures, including the number and the age of the patients visited, the total number of patients that received a given treatment, and notifications about health conditions that require systematic follow-up.

* When GeoHealth was developed the forms were named A, B, C, and D. These forms were redesigned and renamed yet they collect essentially the same data.

Data Analysis & Health Promotion – Running on a web server, GeoHealth-web handles all the data collected by GeoHealth-mobile. This works as a rich repository for health-related action planning used by all level of FHS managers and medical staff. Data analysis supports planning health programs inside a community; prioritize care to critical families; identify epidemic outbreaks, and etc. GeoHealth-web is deployed in a controlled data centre, so that only authorized personnel can access its database. FHTs can recover data from GeoHealth-mobile, being thus able to retrieve information about the families under their responsibility (and only about them). Authorized health managers (e.g. FHTs physicians), on the other hand, can access all data using a web browser, visualizing them as a list of records or as a summary. These users can also plot the georeferenced data with the Google Maps API for easier visualization. Figure 4 illustrates this feature, showing a map for self-reported medical conditions.

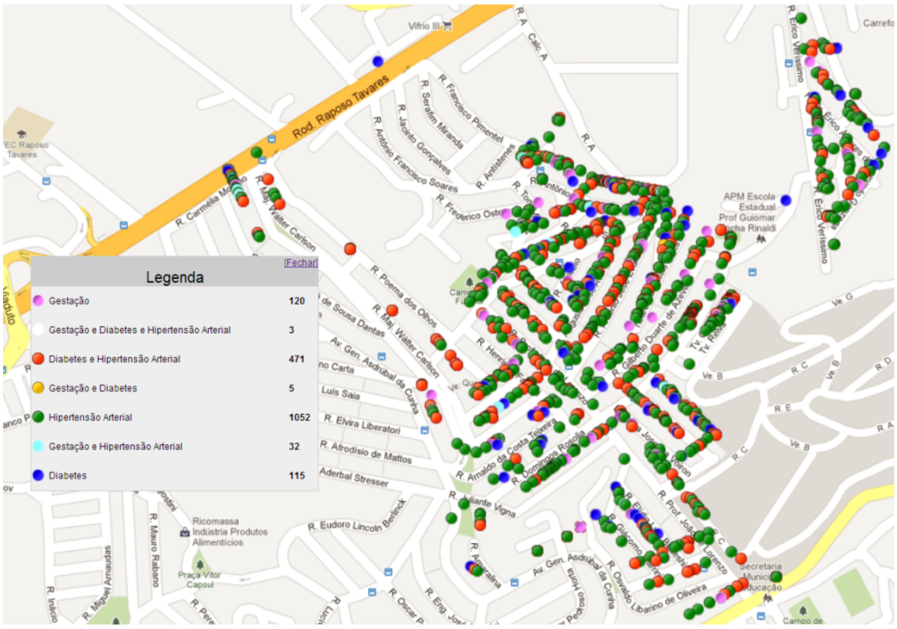

Figure 4. Example of comorbidity map plotted using GeoHealth-web.

Public Health Surveillance – GeoHealth is mainly used by FHTs to act locally, but it should also transmit the collected data to the national database of SIAB, managed by the DATASUS. To do so, GeoHealth-Web has a feature to export data from its database to the SIAB data format. The administrator can thus synchronize databases of GeoHealth and the legacy SIAB software (MS-DOS application). Data transfer to the national SIAB system should be performed through this legacy local SIAB application.

Health-related Research – The GeoHealth database is also a valuable repository for researchers in various fields (e.g. epidemiology, nursing, economy). In fact, members of the FHTs and health managers are often part of academia, making the link between the FHS and university research groups rather strong. However, the GeoHealth system was not designed from the beginning to share data for secondary research purposes. Researchers have to contact FHS managers and system administrators in order to access the data. They would not have access to the database *per se*, but instead, they have to ask the database administrator to run a set of queries and return them the results. Therefore, currently, the access is not automated and requires *ad hoc* agreements to protect families' informational privacy.

Stakeholders – Users and roles in the GeoHealth environment include:

1) *CHAs* that complete surveys on mobile devices;
2) *families* enrolled in the FHS that are visited and surveyed by CHAs;
3) other *FHT members* that access the families' data to provide healthcare;
4) *health managers* (typically part of medical staff) that are responsible for a BHU;
5) *system administrators* that manage the IT infrastructure (e.g. mobile devices, server, databases);
6) *system architects*, an interdisciplinary team of medical professional (e.g. epidemiologists, nurses, physicians) and developers that create the forms and design the system;

7) Brazilian *government agencies* related FHS and UHS (e.g. SIAB and DATASUS); and,
8) *scientists* from public and private universities that may have authorized access to the data for research purposes.

These are the most representative roles in the GeoHealth environment. Users might take one or more roles, and each role might be filled by one or more users.

Data View

The data view aims to identify categories of processed data, data flow diagrams of internal and external flows, including actors and data types. The paper [17] however does not specify how to do it in practice. Our approach therefore uses the implementation guidance provided in the ISO/IEC 29134:2017 [35], i.e. a work flow diagram to visualize PII that is collected and processed by the system.

GeoHealth was designed to replace the paper-based data collection approach and part of the SIAB modules. Once a CHA has finished filling a form during his or her visit, the form is validated by the application, sent to the server and consolidated in the database. CHAs cannot edit or update previously filled forms. The GeoHealth-Mobile saves partially filled forms, but the CHA has to finish filling it during the visit. It is not possible to start a new form if the previous one was not complete. Complete forms are only kept in the mobile's memory card if 3G/4G network is unavailable; otherwise, they are sent directly to the server.

Figure 3 shows how PII is handled by the different sub-processes. The GeoHealth-Web is deployed in a data centre managed by a head institution that coordinates multiple BHUs. The system administrators are also responsible to synchronize the GeoHealth-Web database with the legacy SIAB local module. Originally, each BHU would have a computer running this SIAB module and a member of the medical staff would be responsible to digitize all paper forms into the system. The BHUs would also share a system administrator to manage IT infrastructure and to monthly transmit data from the SIAB local module to the national database, DATASUS. These tasks are now simplified, since all data goes straight to the GeoHealth-Web database and it is easier to export and import it to DATASUS.

Physical Environment View

GeoHealth-Mobile runs in hundreds of smartphones used by the FHS. The devices are shared among CHAs and each BHU has a few dozens of them. CHAs mainly visit families of low- and middle-income levels in urban and rural areas. The protection of the devices (and data) against misuse, lost and theft is a major concern for the project. For this reason, an information security framework named SecourHealth [8] was designed to cope with such issues.

The GeoHealth-Web is deployed in a data centre inside the head institution of the project, i.e. the Heart Institute, a reference hospital part of the University of São Paulo Medical School. Only authorized personnel can enter the site and only system administrators that have credentials to physically access the servers and operate it.
