# Supplementary material for: Mobile Health Systems for Community-Based Primary Care: Identifying Controls and Mitigating Privacy Threats
Source: JMIR Mhealth Uhealth. 2019 Mar 20;7(3):e11642. doi: 10.2196/11642 (PMC6446152; doi:10.2196/11642)
Supplement: Multimedia Appendix 2 [file mhealth_v7i3e11642_app2.pdf]

Multimedia Appendix

This is a Multimedia Appendix to a full manuscript published in the JMIR mHealth and uHealth.

Appendix 2

Evaluation of Degree of Protection Demand

Each of the aforementioned privacy targets should be now put in context and further examined. In this step of the PIA, we are going to rank targets and identify priorities for the GeoHealth's privacy architecture. To determine the right level of protection that each privacy target demands we have to consider a potential damage scenario, i.e, we start by asking "What would happen if...?". Every privacy target should be challenged by its potential damage in case of non-compliance. Furthermore, we have to consider the damage from two perspectives: the system operator (e.g. loss of reputation, financial penalties) and its customer (e.g. social embarrassment, financial losses, jeopardize personal freedom).

Table 5 outlines the strategy used to evaluate the level of protection demanded for each privacy target. A qualitative approach is used because privacy breaches are often "softer" and relate to hurt feelings rather than something with specific monetary value (e.g. a computer system or asset). This approach is also similar to security assessment procedures recommended by the German Federal Office for Information Security (BSI) and the ISO 29134:2017 for PIAs [35].

Table 5. Protection demand categories and perspectives (further details in [18]).

<table>
<tr><td colspan="6">What could be impacted if the privacy target was not met...?</td></tr>
<tr><td colspan="2">System operator perspective</td><td colspan="4">Data subject perspective</td></tr>
<tr><td>Reputation or brand value</td><td>Financial situation</td><td>Social standing, reputation</td><td>Financial situation</td><td>Personal freedom</td><td>Bodily integrity</td></tr>
<tr><td colspan="6">Low – 1: The impact of any loss or damage is limited and calculable. The impact is minimal, acceptable, adverse, for short period, does not endanger personal freedom.</td></tr>
<tr><td colspan="6">Medium – 2: The impact of any loss or damage is considerable. The impact is considerable, seriously adverse, for longer period, could endanger personal freedom.</td></tr>
<tr><td colspan="6">High – 3: The impact of any loss or damage is devastating. The impact is international or nation-wide, threats the existence, devastating, lasting, could seriously endanger personal freedom.</td></tr>
</table>

In what follows, we present an in-depth analysis about the potential damage associated to the privacy targets. To do so, we expand our explanation on each privacy target regarding:

- **"What if...?"** – Potential damages to system operators and customer that can be anticipated.

- **Impact Level** – The estimated level of impact (e.g. low, medium and high) for the different perspectives.
- **Current Situation** – Brief comment about its relevance to the GeoHealth's environment and existing processes of the FHS.

According to the protection demand of each privacy target, we associate one of the three levels of rigour for controls: (1) satisfactory; (2) strong; (3) very strong. For example, high (3) protection demands combined with likely threats should be mitigated with very strong (3) controls, while privacy targets with low (1) impact can be countered with a satisfactory (1) control. As follows, the threat analysis is presented in Appendix C and the recommendation of controls in Appendix D.

P1 - Quality of Data Processing

**P1.1 Ensuring fair and lawful processing through transparency**
**"What if...?"** – If the data processing activities related to the system are not made transparent (internally and externally) to families or other requesting parties 2 , the parties that are involved may face the following consequences:<br><br>- The operator's reputation can be considerably damaged if families are misinformed (e.g. by FHT members) or if they are unable to understand the processing of personal data. The lack of transparency can also be questioned by journalists (i.e. negative press) and authorities (e.g. lawsuits). Disparities between the actual and the expected processing can make families to feel betrayed, increasing distrust in the public healthcare system.<br><br>- The operator's financial situation might be affected by the lack of transparency in cases of negative press and families' adverse reaction to the FHS. If families refrain enrolling in the FHS, in the short term that can entail budget cuts, and in the medium-long term that increases the overall cost of public healthcare due to the lack of preventive actions.<br><br>- Families' reputation is not affected by the lack of transparency per se, but rather by potential violations that may arise when transparency is questioned.<br><br>- Families' financial situation, likewise, is not affected by the lack of transparency *per se*.<br><br>- Personal freedom is seriously damaged when families are forced to enroll to a public healthcare program that is not transparent enough. Many families cannot opt out from the program, since this is often the best (if not only) link between them and the public healthcare system.<br><br>- The integrity of family members is not affected by the lack of transparency per se.<br><br>**Impact Level** – Low. Medium. N/A. N/A. High. N/A.
**Current Situation** – Families receive information about how their data is handled basically through conversation with the FHTs. Currently there is no Transparency Enhancing Tool (TET) in place.

**P1.2 Ensuring processing only for legitimate purposes**
**"What if...?"** – If the purposes are not legitimate (e.g. related to primary care and families treatment) they will be questioned by other parties. Purposes should be made clear to the public in order to assert their legitimacy. If any of the purposes is deemed illegitimate (e.g. discriminatory profiling, surveillance beyond primary care scope), the parties that are involved may face the following consequences:

- The operator's reputation can be seriously damaged in cases of data processing for illegitimate purpose. At worst, a hidden or ill-defined purpose, could be uncovered and lead to public outrage (e.g. if a database is specifically created to monitor people with STDs, or, if housing and health data are cross-reference for income tax analysis). The damage largely depends on how illegitimate a purpose is deemed by the public and authorities.

- Likewise, the operator's financial situation can be seriously affected in cases data processing for illegal purposes (e.g. families refrain using the system leading to budget cuts, lawsuits and fines).

- Families' reputation can be seriously damaged if processing for illegal purposes lead to various kinds of social or economical discrimination.

- Families' financial situation may also be affected since discrimination can lead to social pressure (e.g. lose the job, or move away from the community).

- Data processing for illegitimate purposes can thus severely harm family members' freedom.

- As aforementioned, data processing for illegitimate purposes can potentially lead to discrimination and therefore physical or mental harms to the members of a family.

Impact Level – High. High. High. High. High. High.

Current Situation – The data is not shared with third-parties and all data is processed inside the premises of a reference hospital. This is considered a implied trusted domain with confidentiality among medical staff (i.e. CHAs and health managers must follow strict work policies and regulations). Data is processed to support FHTs and health managers work. GeoHealth-Web just improves the data analysis with more sophisticated interfaces. Data synchronization with DATASUS is still done using the legacy SIAB system.

P1.3 Providing purpose specification

"What if...?" – If the purposes are not adequately specified, they might end up being ambiguous, vague or unlimited. Purposes that are not specific, limited and explicitly defined generate uncertainty. The parties that are involved may face the following consequences:

- The operator's reputation can be adversely affected, since inappropriate purpose specification may reveal incompetence or malicious intent to use of the system. In both cases, that results in bad reputation, public resentment, or negative press.

- The operator's financial situation can be also adversely affected in case of insufficient purpose specification. A complete lack of purpose specification would compromise the whole operation of the FHS. An ambiguous purpose specification may cause families to feel uneasy or refrain using it.

- Families' reputation is not affected by the lack or deficiency of purpose specification per se.

- Families' financial situation is not affected by the lack or deficiency of purpose specification per se.

- Personal freedom is seriously damaged when families are forced to enroll to a public healthcare program that is not clear and understandable enough. Many families cannot opt out from the program, since this is often the best (if not only) link between them and the public healthcare system.

- Family members' integrity is not affected by the lack or deficiency of purpose specification per se.

Impact Level – Medium. Low. N/A. N/A. High. N/A.

Current Situation – Members of the FHTs know that the purpose of GeoHealth is to replace the

paper forms, making the process more efficient and reliable. It is just better tool to do the job. The purpose remains the same. Besides, families have direct access to the FHTs. The CHAs talk about the FHS with the families and orally explain the program. The FHS has been running since 1994, so it is also a well-known among the population. There is plenty of information about it in the governmental websites.

P1.4 Ensuring limited processing for specified purpose
(It should be already limited if P1.2 and P1.3 are followed)

"What if...?" – The processing of data over specified limits gives operators a level of data analysis that is beyond the necessary. The parties that are involved may face the following consequences:

- As in P1.2 and P1.3, operator's reputation can be considerably damaged if unlimited processing is performed over data. This increases the risk of data breaches and privacy invasions (see P6). Besides, the processing of data for reasons (even if justifiable) other than primary care (e.g. secondary purposes, social or economical analysis) would have to be clearly specified. Families are enrolled in a public heath surveillance program, not in a general population census.

- The operator's financial situation can be also considerably damaged in case of unlimited processing. If such operations are uncovered, besides the reputation damage, the program might also loose families and be subject to lawsuits.

- Families' reputation is not directly affected by the processing per se. However, it will be damaged if processing turns out to be illegal (see P1.1), i.e. violating well-specified purposes.

- Families' financial situation is not directly affected by the processing per se.

- Processing beyond limits affects personal freedom of families, since it can potentially allow other kinds of surveillance over a population.

- Family members' integrity is not directly affected by the processing per se.

Impact Level – High. High. N/A. N/A. Medium. N/A.

Current Situation – The GeoHealth is to be used by the FHS (i.e. FHTs and health managers). It is important to keep the purpose of GeoHealth clear and its functions thoroughly documented. In this way it should be clear what the purpose of each function that handles personal data is, thereby preventing any kind of "function creep". This was not done yet.

P1.5 Ensuring data avoidance

Data avoidance is just one strategy for data minimisation. Therefore, we merged this target with the more general one, P1.6 Ensuring data minimisation.

P1.6 Ensuring data minimisation

"What if...?" – If the principles of data avoidance and minimisation are not realised throughout the relevant applications and services, the parties that are involved may face the following consequences:

- The operator's reputation can be adversely affected if families consider that collected data is excessive or unnecessary to provide healthcare. For instance, the GeoHealth should only be used for primary care, so that overly detailed health information is unnecessary and is out of the FHT's scope of treatment. In addition, journalist can question the legitimacy and necessity of collecting excessive amounts of personal data.

- The operator's financial situation can be considerably affected by indiscriminate data collection. It increases the cost of IT infrastructure and the information and notification duties. The greater the

amount of personal data, the greater is the effort to inform and justify families about the system.

- Families reputation is not directly affected by the amount of data collected. However, health data is already sensitive. Excessive collection just increases the damage, if data is misused or leaked.

- Families financial situation is not directly affected by the amount of data collected.

- Family members freedom is not directly affected by the amount of data collected.

- Family members integrity is not directly affected by the amount of data collected.

Impact Level – Medium. High. N/A. N/A. N/A. N/A.

Current Situation – All forms implemented in the GeoHealth were specified by a group composed of physicians, nurses and epidemiologists. All fields have a specific purpose for the primary care and/or public health surveillance. GeoHealth also implements a role-based access control system, so that users can only use functions and handle data that fits their role.

P1.7 Ensuring data quality, accuracy and integrity
(Similar to P7: data integrity)

"What if…?" – If the aspects of data quality (accuracy, up-to-dateness or completeness) are not taken into, the parties that are involved may face the following consequences:

- The operator's reputation can be seriously damaged depending on how serious are the effects of a data errors. Low-quality or incorrect data can lead to wrong decisions in the healthcare support of families (e.g. incorrect treatment or medication). Thus, generating public outrage and negative press. Data quality is crucial for high-quality healthcare.

- The operator's financial situation can be seriously affected depending on the consequences or medical errors due to low data quality. System operators and FHT members can be prosecuted for such mistakes, if families are harmed because of incorrect treatment.

- Families' reputation can be considerably affected if their data is distorted (accidentally or deliberately), e.g. being discriminated by an unfavourable health conditions.

- Families' financial situation can be considerably affected if they do not receive correct treatment from the program, forcing them to move (e.g. to other health units) or pay for treatments that they are entitled to receive for free.

- Families freedom can be adversely affected in case of data distortions that may lead to discrimination or social pressure.

- Family members' bodily integrity can be seriously affected due to incorrect health data. They might not receive appropriate treatment or medication.

Impact Level – High. High. Medium. High. Medium. High.

Current Situation – GeoHealth implements security functions to protect data integrity. All data collected by the CHAs is traceable to a specific user. Access to the database is allowed to system administrators. So, there are means to audit and track the data collection process to identify data distortions and who is accountable for this kind of violation.

P1.8 Ensuring limited storage

"What if…?" – If data is stored longer than necessary and no clear rules are implemented to limit data storage, the parties that are involved may face the following consequences:

- It is very unlikely that the operator's reputation would be damaged by excessive storage of health data.<br><br>- The operator's financial situation can be adversely affected by the increasing cost on storage and management of the IT infrastructure.<br><br>- Families' reputation is not affected by excessive storage of personal data. However, the increasing amount of stored data increases the risk and impact of data breaches and leaks.<br><br>- Families' financial situation is not affected by excessive storage of personal data.<br><br>- Families' freedom is not affected by excessive storage of personal data.<br><br>- Family members' integrity is not affected by excessive storage of personal data.<br><br>**Impact Level** – Low. Medium. Low. Low. Low. Low.
**Current Situation** – GeoHealth currently keeps information about the families in its database even if they move to another city. Currently, there is no agreed procedure on data retention and deletion.

P2 - Processing Lawfulness and Informed Consent

**P2.1 Ensuring legitimacy of personal data processing**
Since GeoHealth processes personal health data (i.e. special category of data, sensitive data), we used only the stricter target P2.2 Ensuring legitimacy of sensitive personal data processing.

**P2.2 Ensuring legitimacy of sensitive personal data processing**
**"What if...?"** – If the legitimacy of processing personal data is not ensured (e.g. no informed consent, not in families' vital interest or public interest), the parties that are involved may face the following consequences:<br><br>- The operator's reputation can be seriously affected for illegitimate data processing, especially because families' personal health data is considered sensitive. Data processing outside the healthcare scope will be criticized by the public and media. For example, the use of family health data by other governmental institutions (e.g. social security, police, tax agency) would likely harm families' privacy, facilitating further profiling and discrimination.<br><br>- The operator's financial situation can be seriously affected by fines and penalties arising from legal actions against the program.<br><br>- Families reputation can be considerably affected, since the processing of health data outside the scope of FHS could lead to false and embarrassing judgments about them.<br><br>- Likewise, families financial situation can be considerably affected, since embarrassment, discrimination and potential false judgments can put them in disadvantaged position, e.g. regarding employability, social and private life.<br><br>- Family members' personal freedom can be also seriously harmed if embarrassment and discrimination leads them to social pressure and exclusion.<br><br>- Family members' bodily integrity can be seriously affected if embarrassment and discrimination generates any kind of mental or physical damage.<br><br>**Impact Level** – High. High. High. High. High. High.

**Current Situation** – GeoHealth was designed to collect and process sensitive data. Families have to enroll the program by giving explicit informed consent, so that the FHS may use GeoHealth to lawfully process their data.

P3 - Information right of data subject (ex ante Transparency)

**P3.1 Providing adequate information in cases of direct collection of data from the data subject**<br>(Strongly related to P1.1)
**"What if...?"** – If adequate information is not provided before direct collection of data, the parties that are involved may face the following consequences:<br><br>- The operator's reputation can be considerably affected for not providing sufficient information about the system. Families might refrain enrolling the program and, in case of misunderstandings, they may feel betrayed. That allows them to withdraw or deny previous informed consent (e.g. claim that they were coerced or that they did not have full knowledge about facts and consequences). This could likely generate negative press about the program.<br><br>- The operator's financial situation can be considerably affected by the lack of transparency, especially if it generates negative press or consumer backlash. The government would have to invest in image campaigns to restore public faith.<br><br>- Families' reputation is not affected by the lack of transparency per se, but rather by potential violations that may arise when transparency is questioned.<br><br>- Families' financial situation, likewise, is not affected by the lack of transparency per se.<br><br>- Personal freedom is seriously damaged when families are forced to enroll to a public healthcare program that is not transparent enough. Many families cannot opt out from the program, since this is often the best (if not only) link between them and the public healthcare system.<br><br>- The integrity of family members is not affected by the lack of transparency per se.<br><br>**Impact Level** – Medium. Medium. N/A. N/A. High. N/A.
**Current Situation** – Although CHAs can provide information about primary care, there is no procedure for explaining about the data processing.

**P3.2 Providing adequate information where data has not been obtained directly from the data subject (e.g. from third parties)**
GeoHealth relies only on data subjects' self-reported health data, and does not collect or process data from third-parties.

P4 - Access right of data subject (ex post Transparency)

**P4.1 Facilitating the provision of information about processed data and purpose**
**"What if...?"** – If no information about processed data (i.e. in the form of data categories and items) and purpose is provided to the consumers, the parties that are involved may face the following consequences:<br><br>- The operator's reputation can be adversely affected by the lack of information about the program, either given orally by FHT members or in writing (e.g. web-sites, booklets and pamphlets). The lack of information may raise suspicion of incompetence or sloppiness, by public and media.

- The operator’s financial situation can be adversely affected if a major part of the population requests detailed reports about their personal data processing.

- Families’ reputation is not affected by the lack of information per se.

- Families’ financial situation is not affected by the lack of information per se.

- Family members’ freedom is not affected by the lack of information per se.

- The integrity of family members is not affected by the lack of transparency per se.

Impact Level – Low. Low. N/A. N/A N/A. N/A.

Current Situation – Regarding the GeoHealth, families are not receiving detailed information about the system. They can however talk if the FHT members that would promptly inform about how their data is used in the FHS and GeoHealth.

P4.2 Facilitating the provision of an (electronic) copy of data
(Related to P5.2)

“What if…?” – If data subjects are not able to receive a copy of their own data, the involved parties may face the following consequences:

- The operator’s reputation can be adversely affected if they cannot provide a copy of data, because data subjects may feel degraded for having their right disrespected. This can also cause public backlash and negative media.

- The operator’s financial situation can be adversely affected in case of possible lawsuits and cost for image campaigns.

- Families reputation is not affected by the absence of data copies.

- Families financial situation is not affected by the absence of data copies.

- Family members freedom is minimally impaired by the absence of data copies, e.g. data subjects are unable request a copy of data before asking for deletion of their information from the system.

- Family members’ integrity is not affected by the absence of data copies.

Impact Level – Low. Low. N/A. N/A. Low. N/A.

Current Situation – Currently there are no procedures to allow data subjects to receive a copy of their data.

P5 - Intervenability

P5.1 Facilitating the rectification, erasure or blocking of data
(Related to P1.7)

“What if…?” – If consumers are not enabled to rectify, erase or block their personal data, the parties that are involved may face the following consequences:

- The operator’s reputation can be adversely affected, especially if families cannot rectify their data. That would lead to low data quality, and therefore, inferior healthcare service.

- The operator’s financial situation can be adversely affected if families cannot rectify their data, e.g.

incorrect data leading to unnecessary health treatments and resource allocation.

- Families reputation can be adversely affected if incorrect data causes them to be mis-categorized with unfavourable health conditions (e.g. that affect employability or carry any kind of social stigma); or if they cannot erase or block personal data, to avoid such categorizations.

- Families' financial situation can be considerably affected if they do not receive correct treatment from the program, forcing them to move (e.g. to other health units) or pay for treatments that they are entitled to receive for free.

- Families freedom can be adversely affected in case of incorrect data that may lead to discrimination or social pressure.

- Family members' bodily integrity can be seriously affected due to incorrect health data. They might not receive appropriate treatment or medication.

Impact Level – Low. Medium. Medium. Medium. Medium. High.

Current Situation – Currently the GeoHealth does not allow data redaction, since forms are consolidated and synchronized with the national SIAB every month. All data about the families is self-reported (i.e. and they can always choose to answer it or not). It should be possible however for families to remove themselves from the GeoHealth database (e.g. right to be forgotten), but there is no process defined for it yet. Besides, the data synchronized with the SIAB should not be deleted (locally or nationally). Changes on the SIAB functions are beyond our capabilities. We are also not considering possible electronic medical record systems that the BHU might have.

P5.2 Facilitating the portability of data

"What if...?" – If data portability is not facilitated, i.e. allowing data transfer from one data controller to another, the involved parties may face the following consequences:

- The operator's reputation can be adversely affected if data managed with GeoHealth is not portable to different systems (e.g. the SIAB, or other potential competitors). The lack of data portability features hampers the system's growth and interoperability. Hence, generating customer dissatisfaction and negative press.

- The operator's financial situation can be seriously affected by the lack of data portability features. The FHS managers should always be able to exchange the GeoHealth system (partially or entirely) for other solutions. Otherwise, the cost of updating or changing the system might become prohibitive in the future.

- Families reputation is not affected by the lack of data portability features.

- Families financial situation is not affected by the lack of data portability features.

- Family members' freedom is minimally affected by the lack of data portability features. If a family moves to another community that is also covered by the FHS, they can re-enroll in the FHS again.

- Family members' integrity is not affected by the lack of data portability features.

Impact Level – Low. High. N/A. N/A. Low. N/A.

Current Situation – GeoHealth is a rather unique system in Brazil, so that data portability would not be very useful after all (i.e. from the customer perspective). If different solutions for the SIAB existed, it would make sense to allow data portability, but mainly to avoid vendor lock-in (i.e. allow system operators to port data from GeoHealth to another system). Other than that, the impact on families' privacy is negligible.

P5.3 Facilitating the notification to third parties about rectification, erasure and blocking of data
Personal data is not shared with third parties other than the public health system itself.

**P5.4 Providing the ability to withdraw consent**<br>(Related to P2.2)
**"What if...?"** – If data subjects are not able to withdraw their consent from the system, the involved parties may face the following consequences:<br><br>- The operator's reputation can be adversely affected if data subjects are unable to withdraw consent. That can allow for lawsuits and negative press, although it seems unlikely.<br><br>- The operator's financial situation could be adversely affected, if they have to pay any fines or invest in image campaigns to regain credibility.<br><br>- Families reputation is not affected by the inability to withdraw consent.<br><br>- Families financial situation is not affected by the inability to withdraw consent.<br><br>- Family members freedom can be adversely affected if they cannot, on any circumstance, withdraw their consent regarding a public health surveillance program. Family members might have good reasons to not be part of such programs, and their privacy and self-determination rights should be respected. If not, they may feel betrayed and uneasy for being within an endless surveillance program.<br><br>- Family members' integrity is not affected by the inability to withdraw consent.<br><br>**Impact Level** – Low. Low. N/A. N/A. Medium. N/A.
**Current Situation** – Families receive visits periodically, allowing them to withdraw consent any time, just by talking to the FHTs. If the consent is withdrawn, the data collection stops, but the previous data is still stored and kept by the system. GeoHealth has no interface that would allow data subjects to do it by themselves.

P6 - Data subject's right to object

P6.1 Facilitating the objection to the processing of personal data
In brief, individuals have the right to object to: (1) processing based on legitimate interests or the performance of a task in the public interest/exercise of official authority (including profiling); (2) direct marketing (including profiling); and, (3) processing for purposes of scientific/historical research and statistics.<br><br>**"What if...?"** – If individuals are not able to raise objections to the processing of their data, the involved parties may face the following consequences:<br><br>- The operator's reputation can be considerably affected if individuals cannot raise objections, even when it is possible to demonstrate that some specific processing overrides their interests, rights and freedoms. In the healthcare, that can generate public dissatisfaction and negative press.<br><br>- The operator's financial situation can be adversely affected in case of possible lawsuits and cost for image campaigns.<br><br>- Family members' social standing cannot be significantly affected.

- Family members’ financial situation cannot be significantly affected.

- Family members’ personal freedom is endangered if they cannot exercise their right to informational self-determination on a relatively large scale. Especially in case of data sharing.

- Family members’ bodily integrity cannot be significantly affected.

Impact Level – Medium. Low. Low. Low. Medium. Low.

Current Situation – Objection of data processing seems rather unlikely to happen with GeoHealth. Currently, we do not know about any type of processing that would fall outside the contract of the FHS.

P6.2 Facilitating the objection to direct marketing activities

Personal is never used for marketing or advertising purposes.

P6.3 Facilitating the objection to disclosure of data to third parties

“What if...?” – If individuals are not able to object the disclosure of their data to third parties (i.e. mainly for research purposes), the parties that are involved may face the following consequences:

- The operator’s reputation can be considerably affected if individuals cannot raise objections, even when it is possible to demonstrate that some specific processing overrides their interests, rights and freedoms. In the healthcare, that can generate public dissatisfaction and negative press.

- The operator’s financial situation can be adversely affected in case of possible lawsuits and cost for image campaigns.

- Families reputation is not directly affected by the loss of the right to object.

- Families financial situation is not directly affected by the loss of the right to object.

- Family members’ freedom is considerably affected if they cannot exercise their right to informational self-determination on a relatively large scale. Once data is shared to a national level (e.g. for research or statistics), they have no means to get it back.

- Family members’ integrity is not directly affected by the loss of the right to object.

Impact Level – Medium. Medium. N/A. N/A. Medium. N/A.

Current Situation – GeoHealth has to share data with SIAB, since this is the legacy system used by the FHS. Besides, the database is also used by researchers. In this case, there is always an ethical approval to be made, and researchers do have direct access to the database. Each case is analyzed separately, and system administrators will normally share de-identified and/or aggregated data to the researchers.

P6.4 Facilitating the objection to decisions that are solely based on automated processing of data

There are no decisions that are made solely based on automated processing of data.

P6.5 Facilitating the data subject’s right to dispute the correctness of machine conclusions

“What if...?” – If individuals are not able to dispute the correctness of personal data that is automatically inferred or collected by the system, the parties that are involved may face the following consequences:

- The operators’ reputation can be minimally impaired since the amount of automatic machine

decisions in very limited and have low impact. Some individuals may request correction but there is virtually no effect on the CHW's main task.<br><br>- The operators' financial situation cannot be significantly affected.<br><br>- Family members' social standing cannot be significantly affected.<br><br>- Family members' financial situation cannot be significantly affected.<br><br>- Family members' personal freedom cannot be significantly affected.<br><br>- Family members' bodily integrity cannot be significantly affected.<br><br>Impact Level – Low. Low. Low. Low. Low. Low.
**Current Situation** – The only data that is automatically collected is the family's location. This information is inferred using GPS and glitches may occur. It would not affected the CHW's visitations but it can affect the upper-level data analysis.

P7 - Security of data

**P7.1 Ensuring the confidentiality, integrity and availability of personal data storage, processing and transmission**<br>(Main references: [21], [22], [23], [24], [43])
Privacy relies on strong security. In short, security measures (technical and organizational) are used to protect data in terms of confidentiality, integrity and availability. Accidental or deliberate attacks to the system can cause data breaches, loss, misuse, and misrepresentation, thus compromising individuals' privacy and safety.<br><br>**"What if…?"** – If security measures fail, the parties that are involved may face the following consequences:<br><br>- The operator's reputation can be seriously affected if security measures fail. Security problems may cause massive data leaks, affecting the entire population covered by the program.<br><br>- The operator's financial situation can be seriously affected in case of security failures. Data loss can be irreversible and recovery may have prohibitive cost. Data breaches may impact privacy of entire communities, leading to public backlash, prosecutions, and negative media.<br><br>- Families reputation can be seriously affected by data breaches, exposing them to all kinds of social embarrassment and pressure.<br><br>- Families financial situation can be seriously affected by security failures. Privacy breaches may have severe social impact on families that may be reflected financially (e.g. loss of job). Data distortions or loss may prevent families from receiving the right treatment and medicine, forcing them to pay for alternative solutions.<br><br>- Family members' freedom is seriously affected by security failures in a public health surveillance system. Entire communities may be profiled and discriminated.<br><br>- Family members' integrity can be seriously affected by security failures, preventing them from receiving appropriate healthcare and aggravating health conditions.<br><br>**Impact Level** – High. High. High. High. High. High.

**Current Situation** – GeoHealth implements a security framework specially designed for mHealth data collection systems. The framework provides all the essential mechanisms for secure data storage, transmission, user authentication and access control.

**P7.2 Ensuring the detection of personal data breaches and their communication to data subjects**
**"What if...?"** – If data subjects are not informed/notified about security incidents related to the compromise of their personal data, the parties that are involved may face the following consequences:<br><br>- The operator's operator's reputation can be considerably impaired because he might get into conflict with the supervisory data protection authority. These conflicts might be exposed to the public.<br><br>- The operator's financial loss can be considerable if he is forced to pay fines, create the necessary documentation ad-hoc with the help of costly consultants and be subject to regular controls by the supervisory authority in the future.<br><br>- Families reputation is not affected by the lack of notification per se.<br><br>- Families financial situation is not affected by the lack of notification per se.<br><br>- Family members' freedom is not affected by the lack of notification per se.<br><br>- Family members' integrity reputation is not affected by the lack of notification per se.<br><br>**Impact Level** – Medium. Medium. N/A. N/A. N/A. N/A.
**Current Situation** – To the best of our knowledge, there is no process defined for incident handling and reporting regarding GeoHealth, SIAB or even the FHS as a whole.

P8 - Accountability

**P8.1 Ensuring the accountability of personal data storage, processing and transmission**
**"What if...?"** – If the system operators are not able fulfill their responsibility to demonstrate compliance with the privacy principles, the parties that are involved may face the following consequences:<br><br>- The operator's reputation can be considerably impaired because he might get into conflict with the supervisory data protection authority. These conflicts might be exposed to the public.<br><br>- The operator's financial loss can be considerable if he is forced to pay fines, create the necessary documentation ad-hoc with the help of costly consultants and be subject to regular controls by the supervisory authority in the future.<br><br>- Families reputation is not affected by the lack of notification per se.<br><br>- Families financial situation is not affected by the lack of notification per se.<br><br>- Family members' freedom is not affected by the lack of notification per se.<br><br>- Family members' integrity reputation is not affected by the lack of notification per se.<br><br>**Impact Level** – High. High. N/A. N/A. N/A. N/A.

**Current Situation** – Since GeoHealth is used for FHS, there are already regulations and medical code of conducts that make FHT members accountable for eventual privacy violations of families' data. GeoHealth has security mechanisms for user authentication, data access control, and logging of users' actions, which facilitate the identification of users responsible for data misuse and leaks. However, a structured privacy audit process remains undefined.

Table 6. Protection demand categories and perspectives.

	System operator perspective		Data subject perspective			
Level/PT	Reputation or brand value	Financial situation	Social standing and reputation	Financial situation	Personal freedom	Bodily integrity
●●● P1.1	Low	Medium	-	-	High	-
●●● P1.2	High	High	High	High	High	High
●●● P1.3	Medium	Low	-	-	High	-
●●● P1.4	High	High	-	-	Medium	-
●●● P1.6	Medium	High	-	-	-	-
●●● P1.7	High	High	Medium	High	Medium	High
●● P1.8	Low	Medium	Low	Low	Low	Low
●●● P2.2	High	High	High	High	High	High
●●● P3.1	Medium	Medium	-	-	High	-
● P4.1	Low	Low	-	-	-	-
● P4.2	Low	Low	-	-	Low	-
●●● P5.1	Low	Medium	Medium	Medium	Medium	High
●●● P5.2	Low	High	-	-	Low	-
●● P5.4	Low	Low	-	-	Medium	-
●● P6.1	Medium	Low	Low	Low	Medium	Low
●● P6.3	Medium	Medium	-	-	Medium	-
● P6.5	Low	Low	Low	Low	Low	Low
●●● P7.1	High	High	High	High	High	High
●● P7.2	Medium	Medium	-	-	-	-
●●● P8.1	High	High	-	-	-	-

* Some Privacy Targets (listed in Table 1) were excluded from the analysis after the protection demand evaluation (i.e. P1.5, P2.1, P3.2, P5.3, P6.2, P6.4). Further information can be found in the Appendix B.
