# Supplementary material for: Mobile Health Systems for Community-Based Primary Care: Identifying Controls and Mitigating Privacy Threats
Source: JMIR Mhealth Uhealth. 2019 Mar 20;7(3):e11642. doi: 10.2196/11642 (PMC6446152; doi:10.2196/11642)
Supplement: Multimedia Appendix 3 [file mhealth_v7i3e11642_app3.pdf]

Multimedia Appendix

This is a Multimedia Appendix to a full manuscript published in the JMIR mHealth and uHealth.

Appendix 3

Identification of Privacy Threats

For each privacy target, we now systematically identify the threats that could prevent us from reaching them. In a PIA, threats are primarily failures to comply with privacy laws or sector standards, which are outlined in the privacy targets. Threats can materialize when stakeholders are ignorant about privacy practices; when technologies do not have adequate privacy functionality; or, when processes and governance practices fails to protect privacy. To ensure a level of methodological control, privacy targets and privacy threats can be matched by using a numbering scheme, as depicted in Figure 5. At last, threats should be marked as "likely" or "unlikely" to exist. If the threat is likely to exist, a control must be determined to mitigate it.

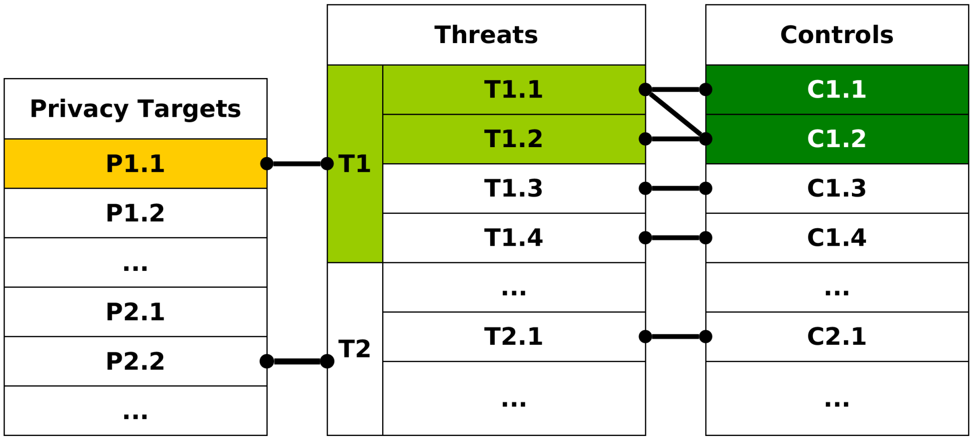

Figure 5. Controlled matching of privacy targets with threats and controls (Adapted from [30]).

As aforementioned, privacy threats have been investigated before, both for mHealth in general [6] or specifically for MDCSs [7], [9]. So that, this threat analysis is based not only on the opinion of privacy experts but also on existing scientific literature in the field.

Considering the work of [7] and [9], the authors mainly focus on security threats (i.e. confidentiality, integrity and availability). These papers present an in-depth analysis regarding security issues, that are related to the principle "P.6 Security of Data", but the identification of privacy threats in general is still largely missing. Similarly, the work of [21] proposes a broader threat taxonomy for mHealth privacy,

categorizing threats in three groups: (a) identify threats; (b) access threats; and, (c) disclosure threats. Although the aim was broader, the resulting threat taxonomy still fails to address many privacy principles (e.g. purpose specification, transparency, data minimisation, accountability, information rights and consent). The taxonomy, therefore, addresses privacy in a "too narrow" way, mainly identifying security-related threats.

In what follows, we list potential threats together with their Associated Privacy Targets (APT) and Likelihood (LH = {**Y**es| **N**o}). Threats that were mentioned in the aforementioned related work are also properly referenced along the text. We are aware that the identification of threats is never complete. This is, however, a joint effort made by the authors to brainstorm and exhaust all relevant privacy threats regarding the GeoHealth system. Furthermore, part of the threats here listed, are also found in the work of [30].

Threats to data quality

**T1 – Lack of transparency – missing or insufficient service information [18]**	APT/LH
**Threat 1.1** – Incomplete or insufficient information describing the service. The operation details (data flows, data locations, ways of transmission, etc) and impacts of GeoHealth are not sufficiently explained to the data subjects.	P1.1 (Y)
**Threat 1.2** – Information on how the system works is denied or difficult to obtain, not easily accessible, or not well-indexed and/or searchable.	P1.1 (Y)
**Threat 1.3** – The basic concept as well as the purpose underlying the service is not clearly explained.	P1.1, P1.3 (N)
**Threat 1.4** – Information is not easy to understand and/or special knowledge is needed to understand, e.g. jurisdictional or technical terminology, institution's internal abbreviations, a distinct language, etc.	P1.1 (Y)
**Threat 1.5** – Information about system changes and its consequences to the families are not kept up-to-date, nonexistent, not clear or insufficient.	P1.1 (Y)

**T1 – Lack of transparency – missing or insufficient privacy statement [18]**	APT/LH
**Threat 1.6** – No privacy statement available.	P1.1 (Y)
**Threat 1.7** – Existing privacy statement does not explain sufficiently how data subject's data is processed.	P1.1 (Y)
**Threat 1.8** – The existing privacy statement does not provide contact information to reach operators and does not provide contact details in case of questions or complaint.	P1.1 (Y)
**Threat 1.9** – The existing privacy statement is difficult to access, i.e. difficult to read, difficult to find, etc.	P1.1 (Y)
**Threat 1.10** – The existing privacy statement does not contain information about relevant third parties that may also receive the data subject's data.	P1.1, P3.1, P4.1 (Y)

**T1 – Unspecified and unlimited purpose [18]**	APT/LH
**Threat 1.11** – Purposes have not been specified or manifested by data controllers before the processing of data starts. It is not specified that the collected data is used only for a distinct purpose or service that is transparent to the data subject as well as to employees.	P1.2, P1.3, P1.4 (Y)
**Threat 1.12** – *Purposes are not specific, limited and explicitly defined.*	P1.3, P1.4 (Y)

**Threat 1.13** – The purpose of the data collection is not documented in an adequate manner.	P1.1, P1.2 (Y)
**Threat 1.14** – Data that is stored and processed only for specific purpose is not marked and/or managed accordingly, e.g. with appropriate access rights.	P1.4 (Y)
**Threat 1.15** – *Data processing does not strictly follow the specified purposes.*	P1.4 (Y)
**Threat 1.16** – *Data processing of families' attributes that may lead to discriminatory profiling.*	P1.2 (Y)
**Threat 1.17** – *Data processing of families attributes that may facilitate mass surveillance unrelated to public healthcare.*	P1.2 (Y)
**Threat 1.18** – *Processing of data for another new purpose that is incompatible to the original ones.*	P1.3, P1.4 (Y)

**T1 – Collection and/or combination of data exceeding purpose [18]**	APT/LH
**Threat 1.19** – *Collected data is inadequate, irrelevant or excessive in relation to the specified purposes.*	P1.5, P1.6 (N)
**Threat 1.20** – *Collected data is not necessary to fulfil overall aim of the processing operations.*	P1.5 (N)
**Threat 1.21** – Processing of data is not logged, thus misuse or processing for another purpose cannot be detected.	P1.2, P1.4 (Y)
**Threat 1.22** – The data subject is required to provide personal data that is not relevant for the specified purpose of the service.	P1.5 (N)
**Threat 1.23** – There are no measures in place that ensure data minimisation. Thus, there are no measures to ensure that only relevant data is processed and that it is not processed excessively in relation to the purpose.	P1.6 (Y)
**Threat 1.24** – There are no measures in place that prevent the linking of data sets. Thus, data collected during the occurrence of the service can be combined with data acquired from a third party or with data from another service the operator/organization is offering.	P1.4, P1.6 (Y)

**T1 – Missing quality assurance of data [18]**	APT/LH
**Threat 1.25** – The identification of the data subject is not conducted thoroughly.	P1.7 (Y)
**Threat 1.26** – *Data is used or processed without reasonable certainty of its accuracy and currentness (up-to-dateness).*	P1.7 (Y)
**Threat 1.27** – Collected data is not properly validated for completeness and correctness before consolidation (e.g. input errors, ambiguous form fields).	P1.7 (Y)
**Threat 1.28** – *Stored data is updated without a legitimate purpose.*	P1.2, P1.4 (N)
**Threat 1.29** – Procedures that regularly check (either by contacting the data subject or automatically searching publicly available data) that data is accurate and up-to-date have not been implemented.	P1.7 (N)
**Threat 1.30** – Personally identifiable data-subject profiles are enriched by probabilistic algorithms that lead to false judgments about a data subject.	P1.7 (N)

**T1 – Unlimited data storage [18]**	APT/LH
**Threat 1.31** – *Personal data is kept stored beyond the time necessary for the purposes for which the data were collected or processed.*	P1.5, P1.8 (Y)
**Threat 1.32** – *Data that is no longer needed is kept stored in a form which permits identification of data subjects (i.e. not de-identified, anonymized, pseudonymized)*	P1.6, P1.8 (Y)
**Threat 1.33** – Data subjects' data as well as corresponding back-up data is not deleted or anonymised when it is no longer needed for the specified purpose. Erasure policies are missing.	P1.5, P1.6, P1.8 (Y)
**Threat 1.34** – Data subjects' data, which is no longer needed for the specified purpose but cannot be deleted due to retention rules, cannot be excluded from regular data	P1.8 (Y)

processing.	

Threats to processing legitimacy & informed consent

**T2 – Invalidation or non-existence of explicit consent [18]**	APT/LH
**Threat 2.1** – *Lack of explicit informed consent of the families.*	P2.1, P2.2 (N)
**Threat 2.2** – *Consent is given without a clear affirmative action (e.g. pre-ticked box, silence).*	P2.1, P2.2 (Y)
**Threat 2.3** – *Data subjects cannot provide consent for a specific purpose (e.g. opt-in for each purpose, only a broad/general consent).*	P2.1, P2.2 (Y)
**Threat 2.4** – *Data controller cannot to prove that consent was given, changed or revoked (i.e. burden of proof).*	P2.1, P2.2 (Y)
**Threat 2.5** – Explicit consent has not been obtained or has been obtained on the basis of incomplete or incorrect information.	P2.1, P2.2 (Y)
**Threat 2.6** – Explicit consent has been obtained based on an offer of advantage or threat of disadvantage.	P2.1, P2.2 (Y)
**Threat 2.7** – The relevant legal basis (e.g. consent, contract, legal obligation, vital interests, public task, balancing interests) has been transgressed.	P2.2 (Y)
**Threat 2.8** – *Denial of processing in case of exemptions, such as: vital interests of the data subject ("break-glass" emergency), legitimate interests of others and public interest.*	P2.2 (N)

Threats to data subject's information right

**T3 – No or insufficient information concerning collection of data from the data subject [18]**	APT/LH
**Threat 3.1** – At the time of data collection, customers are not informed (e.g. orally or in writing, or as a website privacy policy) about: (1) the identity and contact details of the controller; (2) the purpose of the processing; (3) the recipients of the data (e.g. third parties); (4) which questions on the forms are voluntary or optional and what are the consequences of not replying; (5) the existence of the right to access and the right to rectify data.	P3.1, P3.2 (Y)
**Threat 3.2** – The relevant information is not clearly visible (e.g. small pop-up box easily clicked away) at the time of data collection.	P3.1, P3.2 (Y)
**Threat 3.3** – The relevant information is not easy to find but hidden (e.g. small print in the legal section) at the time of data collection.	P3.1, P3.2 (Y)
**Threat 3.4** – Information is not easily understandable by the data subjects, e.g. language is inappropriate for the addressees, excessive or insufficient level of detail.	P3.1, P3.2 (Y)
**Threat 3.5** – The relevant information is not easily understandable; therefore, it is possible that the data subject will not be able to understand that the operator obtained information about him or her from a third party (e.g. location, GPS coordinates).	P3.2 (N)

Threats to data subject's right to access

**T4 – Inability to provide individualised information about processed data and purpose [18]**	APT/LH
**Threat 4.1** – At the time of processing, the operator does not provide any interface to the data subjects to efficiently identify what data about them is processed and what it is used for. Even if the data subject sends a request requiring information, there is no	P4.1 (Y)

procedure to automatically obtain this individualised information from the operator's system.	
**Threat 4.2** – Access is possible, but not to all relevant data, including: (1) confirmation as to whether or not data relating to the data subject is being processed; (2) the purpose of processing; (3) the categories of data concerned; (4) the recipients or categories of recipients to whom the data is disclosed; (5) the data undergoing processing and any information as to the data's source; (6) the logic involved in any automatic processing of data and automated decisions.	P4.1 (Y)
**Threat 4.3** – The identity of the data subject is not or not sufficiently checked (i.e. insufficient authentication) before allowing access.	P4.1, P7.1 (Y)
**Threat 4.4** – Successful access as well as subsequent data disclosure is not logged.	P4.1, P7.2 (Y)

Threats to intervenability

**T5 – Inability to rectify, erase or block individual data [18]**	APT/LH
**Threat 5.1** – There is no implemented procedure (technical and/or processes) that allows the data subject to rectify, erase or block individual data.	P5.1 (Y)
**Threat 5.2** – *Data subjects cannot have their data deleted or blocked by the controller, in case of illegal processing (e.g. consent withdrawn, data no longer needed to fulfil the purposes).*	P5.1, P5.4 (Y)
**Threat 5.3** – Errors are not automatically rectified.	P1.7, P5.3 (Y)
**Threat 5.4** – There is no procedure that allows the erasure of individual data in back-up data.	P5.1 (Y)
**Threat 5.5** – The identity of the data subject is not or not sufficiently checked (insufficient authentication) before rectification, erasure or blocking of data.	P5.1, P7.1 (Y)
**Threat 5.6** – Successful rectification, erasure and blocking is not logged.	P5.1, P8.2 (Y)

**T5 – Inability to notify third parties about rectification, erasure and blocking of individual data [18]**	APT/LH
**Threat 5.7** – The operator has not implemented any procedure to notify relevant third parties when individual data has been rectified, erased or blocked.	P5.3 (Y)

T5 – ***Inability to support data portability for individual data***	APT/LH
**Threat 5.8** – *Data subjects cannot obtain a portable version of their personal data in a structured, commonly used and machine-readable format.*	P5.2 (Y)
**Threat 5.9** – *Data subjects cannot request a direct transmission of their data to another agency (where technically feasible).*	P5.2 (Y)
**Threat 5.10** – *Data subjects cannot request an individualised (electronic) copy of their own data.*	P4.2 (Y)

Threats to data subject's right to object

**T6 – Inability to allow objection to the processing of personal data [18]**	APT/LH
Threat 6.1 – There is no implemented procedure (technical and/or processes) that allows the data subject to object to the processing of personal data (based on compelling legitimate grounds relating to the data subject's particular situation).	P6.1 (Y)

T6 – Inability to allow objection to the disclosure of data to third parties	APT/LH
**Threat 6.2** – *The right to object is not raised to data subjects before data are made available.*	P6.3 (Y)
**Threat 6.3** – *Data subject does not have the opportunity to object before data disclosures occur.*	P6.3 (Y)
**Threat 6.4** – *The data subject is not informed about the disclosure of his data to third parties and thus the data subject cannot object.*	P6.3 (Y)
Threat 6.5 – *The operator has not implemented any procedure to notify relevant third parties when a data subject has objected to the processing of his personal data.*	P5.3, P6.3 (Y)

Threats to data security

**T7 – Identity threats: misuse and leakage of data subject identities [6], [41]**	APT/LH
**Threat 7.1** – Insiders (e.g. FHT members or medical staff) misuse data subject's identities and input fake data to avoid doing the visitations (identity theft and forgery).	P7.1 (Y)
**Threat 7.2** – Outsiders can re-identify individuals and their personal data in de-identified data sets.	P1.6, P7.1 (Y)
**Threat 7.3** – Outsiders can eavesdrop the communication channel to observe data subject's identity and/or location.	P7.1 (Y)

**T7 – Access threats: unauthorized access and modification of PHI or PHR [6], [41]**	APT/LH
**Threat 7.4** – Data subjects fail to express their consent consistently with their actual preference, for whatever reason, allowing broader than intended collection, access or disclosure.	P7.1 (Y)
**Threat 7.5** – Insiders access data subject's data by mistake due to over-privilege or inadequate controls.	P1.4, P1.6. P7.1 (Y)
**Threat 7.6** – Insiders modify data subject's data by mistake due to over-privilege or inadequate controls.	P1.4, P1.6. P7.1 (Y)
**Threat 7.7** – Insiders commit intentional unauthorized access, for their personal benefit (e.g. bribing, curiosity or malice) or due to external coercion.	P7.1 (Y)
**Threat 7.8** – Insiders commit intentional modifications, for their personal benefit (e.g. bribing, curiosity or malice) or due to external coercion.	P7.1 (Y)
**Threat 7.9** – Outsiders commit intentional unauthorized access, for curiosity or malice.	P7.1 (Y)
**Threat 7.10** – Outsiders commit intentional modification, for fraud or malice.	P7.1 (Y)
**Threat 7.11** – Outsiders demand legal access to the data, e.g. through a subpoena.	P2.1, P2.2, P7.1 (Y)

**T7 – Disclosure threats: unauthorized disclosure and data leaks of PII and PHI [6], [41]**	APT/LH
**Threat 7.12** – Data at rest (server-side) – Insiders accidentally disclose personal data due to misconfigurations in the GeoHealth data sharing modules.	P7.1 (N)
**Threat 7.13** – Data at rest (server-side) – Insiders accidentally disclose personal data due to improper password sharing among the staff.	P1.4, P1.6, P7.1 (Y)
**Threat 7.14** – Data at rest (server-side) – Insiders intentionally disclose personal data for profit or malice.	P7.1 (Y)
**Threat 7.15** – Data at rest (server-side) – Outsiders intentionally disclose personal data for profit or malice.	P7.1 (Y)
**Threat 7.16** – Data at rest (mobile-side) – Insiders accidentally disclose personal data due to malwares and mis-configured devices, e.g. fake application installed, malicious applications installed on devices, data leaking to other applications.	P7.1 (Y)
**Threat 7.17** – Data at rest (mobile-side) – Loss or theft of mobile devices, allowing outsiders to expose personal data, credentials and key materials.	P7.1 (Y)

**Threat 7.18** – Data at rest (mobile-side) – Improper disposal of devices used in data collection, allowing outsiders to expose personal data, credentials and key materials.	P7.1 (Y)
**Threat 7.19** – Data in transit – Outsiders eavesdrop the communication channels between mobile devices and server for traffic analysis and/or content decryption.	P7.1 (Y)

**T7 – Denial-of-service threats [7], [9]**	APT/LH
**Threat 7.20** – Inability to use the data collection application, e.g. the application gets misconfigured, lack of network connectivity (unable to authenticate and transmit data).	P7.1 (Y)
**Threat 7.21** – Denial-of-service attacks preventing data from being up-loaded to the server, e.g. outsiders disrupts the communication channel, device or server functions.	P7.1 (Y)

**T7 – Inability to detect of personal data breaches and communicate them to data subjects [18]**	APT/LH
**Threat 7.22** – Failing to detect infringements of data security in the system (e.g. no internal procedures for management and reporting of data breaches).	P7.2 (Y)
**Threat 7.23** – Failing to notify data breaches to the likely victims (i.e. data subjects).	P7.2 (Y)
**Threat 7.24** – Failing to notify data breaches to the competent authorities.	P7.2 (Y)

Threats to accountability

**T8 – Lack of accountability of personal data storage, processing and transmission [18]**	APT/LH
**Threat 8.1** – Controllers fail to implement the measures to promote and safeguard data protection in their processing activities.	P8.1 (Y)
**Threat 8.2** – Controllers are not able to demonstrate (at any time) compliance with data protection provisions to data subjects, to the general public and to supervisory authorities.	P8.1 (Y)

Threats to notification/reporting duties

In addition to application-related threats, system operators should also be aware of their *reporting duties*. So that, the following “threats” are relevant to operators in order to comply with the privacy regulation (i.e. GDPR).

**T8 – Non-compliance with notification requirements [18]**	APT/LH
**Threat 8.3** – The operator does not notify the supervisory authority or the internal data protection officer as legally defined before carrying out personal data processing.	P8.1 (Y)
**Threat 8.4** – The operator does not provide all the legally defined contents in his notification to the supervisory authority or the internal data protection officer.	P8.1 (Y)
**Threat 8.5** – The operator does not publish or does not ensure the availability of the legally defined notification contents to any person on request.	P8.1 (Y)
**Threat 8.6** – The operator does not ensure the availability of the PIA report six weeks before the launch or upgrade of the system (i.e. GeoHealth) application.	P8.1 (Y)
