# Supplementary material for: Mobile Health Systems for Community-Based Primary Care: Identifying Controls and Mitigating Privacy Threats
Source: JMIR Mhealth Uhealth. 2019 Mar 20;7(3):e11642. doi: 10.2196/11642 (PMC6446152; doi:10.2196/11642)
Supplement: Multimedia Appendix 4 [file mhealth_v7i3e11642_app4.pdf]

Multimedia Appendix

This is a Multimedia Appendix to a full manuscript published in the JMIR mHealth and uHealth.

Appendix 4

Technical and Non-Technical Controls

Here we use the list of controls presented in [30] as well as the security controls proposed in [7], [8], [9], [41]. In [30], the authors composed a list of 26 control measures that can minimise, mitigate or eliminate the identified privacy threats (see Appendix C for further details). Each control has up to three levels of rigour: (1) satisfactory; (2) strong; and, (3) very strong. So that, during the process of selecting controls for each threat, we also choose its level of rigour that defines how extensive (likely more costly and difficult) it should be. The level of rigour should match the previously defined level of protection demand determined in Section "Evaluation of Degree of Protection Demand for Each Privacy Target".

Control descriptions

It is possible to realise that there is no control associated exclusively to a 'Low' or 'Medium' threat. Most controls are associated to more than one threat, so that the level of rigour should be always rounded up to the most critical threat. As a result, for GeoHealth all the controls have a 'High' level of rigour. Their descriptions are the following.

**C1.1 Service Description [18] (T1.1, T1.3, T1.4)**
Extensive informational material (e.g. flyers, websites) is made available that is easily understandable and accessible. The technical functionality of the GeoHealth technology is explained. Information about data processing, such as data flows, data location, and methods of transmission, is described in detail.

**C1.2 Information Accessibility [18] (T1.2)**
The information describing the service is proactively provided to the data subjects. It is made available in such a way that the data subject's attention is attracted. Online content is well-indexed and searchable.

**C1.3 Language/Semantics of Information [18] (T1.4)**
The information describing the service is available in the language of the operator's home country and does not contain any expression requiring special knowledge (such as jurisdictional knowledge or company-internal terminology).

**C1.4 Information Timeliness [18] (T1.1, T1.5)**
(1) Each time there are changes to the service and the underlying application, the information describing the service is updated accordingly. (2) The information describing the service is checked at regular intervals for its timeliness, especially when questions from data subjects could not be answered.

C1.5 Privacy Statement [18] (T1.6, T1.7, T1.8, T1.9, T1.10)

An extensive privacy statement that contains all required information in an easily understandable form is easily accessible, i.e. prominently linked from each web page of the operator. It is available in the most common languages.

C1.7 Purpose Specification [18] (T1.11, T1.12, T1.13)

A purpose specification is available in two versions: a very detailed version for the involved employees of the operator that includes system and application details and a version that is written for the data subjects. The latter one is easily accessible, e.g. on the operator's website. The former one is regularly brought to the attention of the employees, e.g. during privacy training, to increase their awareness.

C1.8 Ensuring Limited Data Processing [18] (T1.14, T1.16, T1.17, T1.24)

Collected data is secured with access rights that correspond to the specified purpose. Access rights can be specified on a fine-grained level.

C1.9 Ensuring Purpose Related Processing [18] (T1.15, T1.16, T1.17, T1.18, T1.21)

It is regularly checked that collected data is used only for the specified purpose. Corresponding access rights are regularly checked and updated. Access to data and processing of data is logged on a level that is sufficient to detect potential misuse or processing for another purpose than the specified one.

C1.10 Ensuring Data Minimisation [18] (T1.16, T1.17, T1.19, T1.20, T1.22, T1.23, T1.33)

Data collection is regularly checked under the aspect of data minimisation (e.g. storage avoidance, minimal granularity). Thus, it is regularly questioned whether only relevant data (relevant to the specified purpose) is collected from data subjects. Additionally, there are technical procedures in place, which ensure data minimisation (e.g. in particular automated enforcement of deletion policies, implementation of anonymisation, pseudonymisation and obfuscation) during processing of data.

C1.12 Ensuring Personal Data Quality [18] (T1.27)

Data collection forms and tools are designed and implemented in such a way that completeness and correctness of the data collected from data subjects can be ensured in the best possible way.

C1.14 Ensuring Data Accuracy [18] (T1.26, T1.28, T1.29, T1.30)

Technical procedures are in place that automatically ensure that data is accurate and up-to-date, e.g. by searching through publicly available data or regularly asking all data subjects to check and rectify their data.

C1.15 Enabling Data Deletion [18] (T1.31, T1.32, T1.33, T1.34, T5.1)

Data subjects' data that is no longer needed for the specified purpose is deleted or anonymised. Corresponding data in back- up systems is deleted, too. Legal retention requirements are considered. Data that cannot be deleted due to retention rules is marked as such and excluded from regular data processing.

C3.1 Obtaining Data Subject's Explicit Consent [18] (T2.1, T2.2, T2.3, T2.4, T2.5, T2.6, T2.7, T2.8)

Legal personnel regularly check if necessary explicit consent is obtained at all and whether it is obtained on the basis of complete or correct information and not upon an offer of advantage or threat of disadvantage. Consent forms and the like are checked by legal personnel. Rules/policies concerning the legitimacy of processing sensitive personal data have been described and are available to all employees of the operator. Additionally, employees are taught about this subject

during regular privacy training sessions to increase their awareness.

C4.1 Providing Data Processing Information [18] (T3.1, T3.2, T3.3)

At the time of data collection, the data subject has access to information that describes all relevant data:

- the identity of the data controller and of his representative if any,
- the purpose of the processing,
- the recipients of the data (is the data given to any third party?),
- which questions on the registration form are voluntary and which are optional and what are the consequences of not replying,
- the right to access and rectify the data about him.

For example, this information is explicitly and easily understandable, and is presented and integrated into the data collection form or tool.

C4.2 Providing Information on Third Party Information Processing [18] (T3.1, T3.5)

When data is obtained from a third party, the data subject has access to information that describes all relevant data:

- the identity of the data controller and of his representative if any,
- the purpose of the processing,
- the categories of data concerned,
- the recipients of the data (is the data given to any third party?),
- the existence of the right of access to and the right to rectify the data concerning him.

E.g. this information is explicitly provided to him in an easily understandable way.

C5.1 Informing Data Subjects About Data Processing [18] (T3.4, T3.5, T4.1, T4.2)

There is an application available to every data subject that enables him or her to efficiently get information about his or her processed data. In particular:

- confirmation as to whether or not data relating to the data subject is being processed,
- the purpose of the processing,
- the categories of data concerned,
- the recipients or categories of recipients to whom the data is disclosed,
- the data undergoing processing and any information as to the data's source,
- the logic involved in any automatic processing of data and automated decisions.

Requests are automatically processed and individualised information is retrieved from the operator's systems.

C5.3 Handling Data Subject's Change Requests [18] (T5.1, T5.2, T5.3, T5.4, T5.7)

There is an application available to every data subject that enables him or her to efficiently request and conduct rectification, erasure or blocking of his or her processed data. Requests are automatically processed and individualised operations are performed in the operator's systems. In the case of data erasure, relevant data in backup systems is erased too. When data is changed that is relevant for third parties, a notification is sent out that describes the changes.

C5.4 Providing Data Export Functionality (T5.8, T5.9, T5.10)

There is an interface that allows data subjects to download and/or transmit their personal data. Data portability features include:

- Export/download electronic copy of personal data.
- Employ machine-readable portable formats and data standards.
- Direct transmission of personal data among service providers (if possible in the business scenario).

C5.5 Handling Exemptions and Derogations (T2.8, T7.11)

There are exemptions from the GDPR's transparency obligations and individual rights, but only where the restriction respects the essence of the individual's fundamental rights and freedoms and is a necessary and proportionate measure in a democratic society to safeguard. Such exemptions should be identified and procedures should be put in place to handle these situations. Examples include to carry out a task of public interest, or when processing is in the vital interest of data subjects (e.g. break-the-glass mode in health systems), or to deal with data access attempts through legal demands (e.g. subpoena).

**C6.1 Notifying Data Subjects about Sharing Practices [18] (T6.1, T6.4)**
Notifications are sent to the data subject whenever the operator plans to disclose data to third parties or to use data for a purpose that has not been explicitly stated before, such as for direct marketing. There is an application available to every data subject that enables him or her to efficiently create objections. Requests are automatically processed and individualised operations are performed in the operator's systems. In the case of involved third parties a notification is sent out to relevant third parties.

**C6.2 Handling Objections to Automated Decisions [18] (T1.1, T6.1, T6.2, T6.3, T6.6)**
(1) The logic involved in any automatic processing of data and automated decisions is described and made available to the data subjects. They are informed of their right to object to this automated decision making. A contact address is given. Objections are individually processed and automated decisions are disabled on request. (2) There is an application available to every data subject that enables him or her to access detailed information about the automated decision procedures that are used and to object to these or even alter / influence them. Objections are automatically processed and automated decisions are disabled.

C7.1 Ensuring Data Subject Authentication (T1.25, T4.3, T5.5, T7.1)
The data subject needs to identify or authenticate himself with a valid ID or eID, either personally or online.

C7.2 Ensuring Staff Authentication (T7.9, T7.13)
The healthcare workers have to authenticate themselves to use the GeoHealth system on the smartphones or in the web browser.

C7.3 Ensuring Device Authentication (T7.16, T7.21)
The smartphones have to authenticate themselves with the GeoHealth-Web server (and/or the MNO) to ensure that only properly configured devices are allowed to transmit data.

C7.4 Providing Usable Authentication (T7.13)
The adopted authentication mechanisms have to observe usable security principles and usability aspects.

C7.5 Logging Access to Personal Data (T4.4, T5.6, T7.1, T7.5, T7.6, T7.7, T7.8, T7.12, T7.13, T7.14, T7.15, T7.16, T7.22)
Data subjects' access to data, subsequent data disclosure, rectification, erasure and blocking are logged on a level that is sufficient to ensure accountability.

C7.6 Performing Regular Privacy Audits (T7.1, T7.7, T7.8, T7.12, T7.13, T7.14, T7.15, T7.22, T8.1, T8.2)
The operator, system administrators and project managers needs to carry out regular privacy audits to ascertain its privacy practices and pro-actively detect violations.

C7.7 Ensuring Data Anonymisation (T7.2)

The operator has to employ anonymisation techniques that prevent the re-identification of data subjects, having regard to all means "likely reasonably" to be used for re-identification (by the operator itself, third-parties or adversaries).

C7.8 Providing Confidential Communication (T7.3, T7.19)
The operator has to employ mechanisms to secure the confidentiality of the communication and data transmission between smartphones and servers (e.g. encrypted channels).

C7.9 Providing Usable Access Control (privacy preferences) (T7.4, T7.5, T7.6)
The adopted access control mechanisms have to observe usable security principles and usability aspects.

C7.10 Ensuring Secure Storage (T7.9, T7.10, T7.17, T7.18, T7.20)
The operator has to employ mechanisms to secure the confidentiality of the data stored in the smartphones (e.g. data encryption).

C7.11 Ensuring Physical Security of Infrastructure (T7.9, T7.10)
The operator needs to adopt security measures to deny unauthorized access to facilities (e.g. BHUs), equipment and resources (e.g. smartphones and servers) and to protect them from damage or harm.

C7.12 Providing Locked Down Devices (T7.16)
The operator needs to apply to the smartphones a lock down configuration that allows only the use of authorised applications and functions.

C7.13 Providing Memory Wipe (T7.17, T7.18)
The operator needs to employ mechanisms for memory erasure in case of compromised smartphones. The smartphone should be able to wipe its memory automatically in case of specific security violation, or, be remotely wiped by commands from a system administrator.

C7.14 Enabling Offline Authentication (T7.20)
The operator needs to provide a secondary offline mechanism for authenticating data subjects and healthcare workers during the data collection process. The application should be operational even when there is a lack of connectivity (i.e. mobile network).

C7.15 Network monitoring (T7.21)
The operator needs to consistently monitor its network infrastructure in order to prevent or detect attacks. (Not sure if we should keep.)

C7.16 Preventing DoS attacks (T7.21)
The operator needs to employ security measures to prevent DoS/DDoS attacks to its IT infrastructure (e.g. firewalls, white-list smartphones, IPS-based prevention).

C7.17 Handling Security Incidents (T7.22)
The operator needs to employ procedures for an organised and careful reaction to privacy and security incidents (e.g. privacy violations and data breaches).

C8.1 Demonstrate Data Privacy Accountability (T8.1, T8.2)
Integrate accountability as a principle which requires that organisations put in place appropriate technical and organisational measures and be able to demonstrate what they did and its effectiveness when requested. Organisations, and not Data Protection Authorities, must demonstrate that they are compliant with the law. Such measures include:

- adequate documentation on what personal data are processed, how, to what purpose, how long;
- documented processes and procedures aiming at tackling data protection issues at an early state when building information systems or responding to a data breach;
- the presence of a Data Protection Officer that be integrated in the organisation planning and operations etc.

Additionally, organisations can also adopt Privacy Management Tools and Privacy Maturity Models.

C8.2 Notification of Authority [18] (T7.24, T8.3, T8.4, T8.6)

It is ensured that the supervisory authority is notified before going live with the GeoHealth application and that this notification contains all required information about the personal data processing. In addition, the PIA report needs to be made available to the authorities at least 6 weeks prior to the GeoHealth applications launch.

C8.3 Notification of Data Subjects [18] (T7.23)

It is ensured that data subjects are informed when changes in the system can impact their privacy or when there data breaches happen.

C8.4 Prior Checking [18] (T8.5)

It is ensured that the legally required checking of the GeoHealth application is executed by expert personnel.
